# PTP1B deficiency in myeloid cells increases susceptibility to *Candida albicans* systemic infection by modulating antifungal immunity

**DOI:** 10.1128/mbio.01516-25

**Published:** 2025-08-29

**Authors:** Bethany Allen, Ivy M. Dambuza, Susan H. Berry, Delyth M. Reid, Sam M. McVey, Martina Mesiarikova, Larissa John, Moira Davie, Christa P. Baker, J. Simon C. Arthur, Mirela Delibegovic, Gordon D. Brown, Heather M. Wilson

**Affiliations:** 1 School of Medicine, Medical Sciences & Dentistry, Institute of Medical Sciences, University of Aberdeen 1019, Aberdeen, United Kingdom; 2 MRC Centre for Medical Mycology, University of Exeter 3286, Exeter, United Kingdom; 3 Department of Pathology, Aberdeen Royal Infirmary 59633, Aberdeen, United Kingdom; 4 Division of Cell Signalling and Immunology, School of Life Sciences, University of Dundee 3042, Dundee, United Kingdom; 5 Aberdeen Cardiovascular and Diabetes Centre, University of Aberdeen 1019, Aberdeen, United Kingdom; University of Florida College of Dentistry, Gainesville, Florida, USA

**Keywords:** macrophages, *Candida albicans*, protein tyrosine phosphatase 1B, infections

## Abstract

Invasive candidiasis, primarily caused by *Candida albicans*, poses a significant threat to immunocompromised patients, with high mortality rates. Understanding how immune responses to *Candida albicans* are mounted and controlled is fundamental to developing new therapeutic strategies. Protein tyrosine phosphatase 1B (PTP1B) is a regulator of immunoreceptor signaling and downstream inflammatory and metabolic responses and a pharmaceutical target. Here, we reveal a critical role for myeloid cell-intrinsic PTP1B in antifungal immunity. Mice lacking PTP1B in myeloid cells (LysM PTP1B^−/−^) were significantly more susceptible to systemic *C. albicans* infection, exhibiting lower survival, greater weight loss, and elevated fungal burdens in the kidney, liver, and brain. These mice also showed heightened proinflammatory mRNA expression in organs and increased kidney tubular inflammation, with increased leukocyte infiltration and chemokine production, contributing to immunopathology. Neutrophils from LysM PTP1B^−/−^ mice displayed impaired maturation in infected kidneys and reduced reactive oxygen species production *in vitro*. Proteomic profiling of infected bone marrow-derived macrophages (BMDMs) revealed significant enrichment of type I interferon-regulated proteins in the absence of PTP1B. These BMDMs showed impaired phagocytosis, reduced killing capacity, and lower viability during infection, phenotypes recapitulated in human macrophages treated with a pharmacological PTP1B inhibitor. Collectively, our findings highlight PTP1B as a key modulator of innate immune responses to *C. albicans*, balancing antifungal activity and systemic toxicity with inflammation and metabolic fitness. Boosting specific PTP1B-dependent pathways may offer new strategies for enhancing host defense while minimizing fungal-induced immunopathology.

**IMPORTANCE:**

Systemic *Candida albicans* infections are a leading cause of hospital-acquired morbidity and mortality, particularly in immunocompromised individuals and patients receiving immunosuppressive treatments. Despite antifungal therapies, outcomes remain poor, underscoring the need to better understand host factors that control fungal clearance. Protein tyrosine phosphatase 1B (PTP1B) is a key intracellular regulator of immune and metabolic signaling. This study identifies a critical role for myeloid PTP1B in antifungal defense and susceptibility to systemic *C. albicans* infection. Loss of PTP1B impairs neutrophil and macrophage function, disrupts inflammatory balance, and compromises pathogen clearance. These findings reveal PTP1B as a central modulator of immune responses to *C. albicans* and highlight its potential as a target for host-directed therapies to improve outcomes in systemic fungal infections.

## INTRODUCTION

Invasive fungal infections are a significant clinical burden, especially for immunocompromised patients and those undergoing clinical procedures such as chemotherapy, surgery, or organ transplantation. There are over 3.8 million deaths globally per year associated with fungal infections (1). The *Candida* species are the most common fungal pathogens responsible for opportunistic infections, and *Candida albicans* is the most widespread pathogen associated with nosocomial infections (1, 2). The growing number of invasive fungal infections worldwide and the increasing numbers of at-risk patients and their hospitalization, alongside increasing resistance to antifungal drugs, have highlighted the need to better understand the mechanisms of host immune defense to candidiasis. This particularly relates to the pathways regulating immune cell responses following infection, and insights into this are key for developing new antifungal drugs to improve clinical outcome (3).

The initial host response to *C. albicans* infection is activation of innate immune cells, neutrophils, monocytes/macrophages, and dendritic cells, through recognition of fungal pathogen-associated molecular patterns by host pathogen recognition receptors (PRRs) that elicit antifungal defenses (4). Key PRRs in fungal recognition are Toll-like receptors (TLRs), C-type lectin receptors, and nucleotide oligomerization domain-like receptors, which, through kinases and phosphatases, induce phosphorylation and dephosphorylation of signaling molecules as fundamental immune cell regulatory mechanisms (5). One phosphatase that has been shown to have a regulatory role in several important pathways in immune cell responses is the non-receptor protein tyrosine phosphatase 1B (PTP1B) (6). PTP1B has multiple substrates, many of which are receptors that utilize tyrosine kinases for signaling, such as cytokine receptors, and the insulin and leptin receptors (6, 7). PTP1B regulates these targets via direct dephosphorylation or regulation of receptor endocytosis (6, 7). Because of its role in dephosphorylating the insulin receptor and controlling cell proliferation, PTP1B is regarded as an attractive target for the treatment of diabetes, obesity, and some cancers, and inhibitors have progressed to clinical trials (6, 8).

PTP1B expression is strongly upregulated in immune cells upon pro- or anti-inflammatory challenge, and as Janus kinase 2 (Jak2), tyrosine kinase 2 (Tyk2), and colony stimulating factor 1 receptor (CSF1R) receptor are substrates of PTP1B, innate immune signaling can be significantly impacted (9–13). Moreover, PTP1B-deficient macrophages and neutrophils show alterations in key signaling pathways involved in immune processes, including regulatory networks involving MyD88, TRIF, IRF3, STAT1, STAT3, and STAT6 (9–12, 14, 15). Previous studies have highlighted an important role of PTP1B in regulating overall immune cell responses toward bacterial and viral infection (12, 14, 16–18). For example, PTP1B negatively impacts host defense against *Pseudomonas aeruginosa* infection and mediates lung damage during respiratory syncytial virus exacerbations (12, 16). However, the role of PTP1B in immune cell defense during fungal infection and the mechanisms it controls in this process remain to be established.

In this study, we deployed cellular, biochemical, proteomic, metabolic, and molecular approaches using myeloid-specific PTP1B knockout mice and isolated murine and human immune cells to address the question of how PTP1B deficiency or inhibition can alter the susceptibility to systemic *C. albicans* infection. We demonstrate that PTP1B functions as a critical negative regulator to limit systemic fungal infection and associated inflammation and propose a new mechanism whereby PTP1B modulates immune cell functions during host defense against *C. albicans* through its effects on phagocytosis, killing efficacy, and rewiring macrophage metabolism to influence cell viability. Overall, this study expands our understanding of defense mechanisms against fungal infection with therapeutic implications for controlling innate immune responses.

## RESULTS

### Loss of myeloid cell PTP1B upregulates systemic *C. albicans* **i**nfection and inflammatory responses in mice

**Fig 1:**
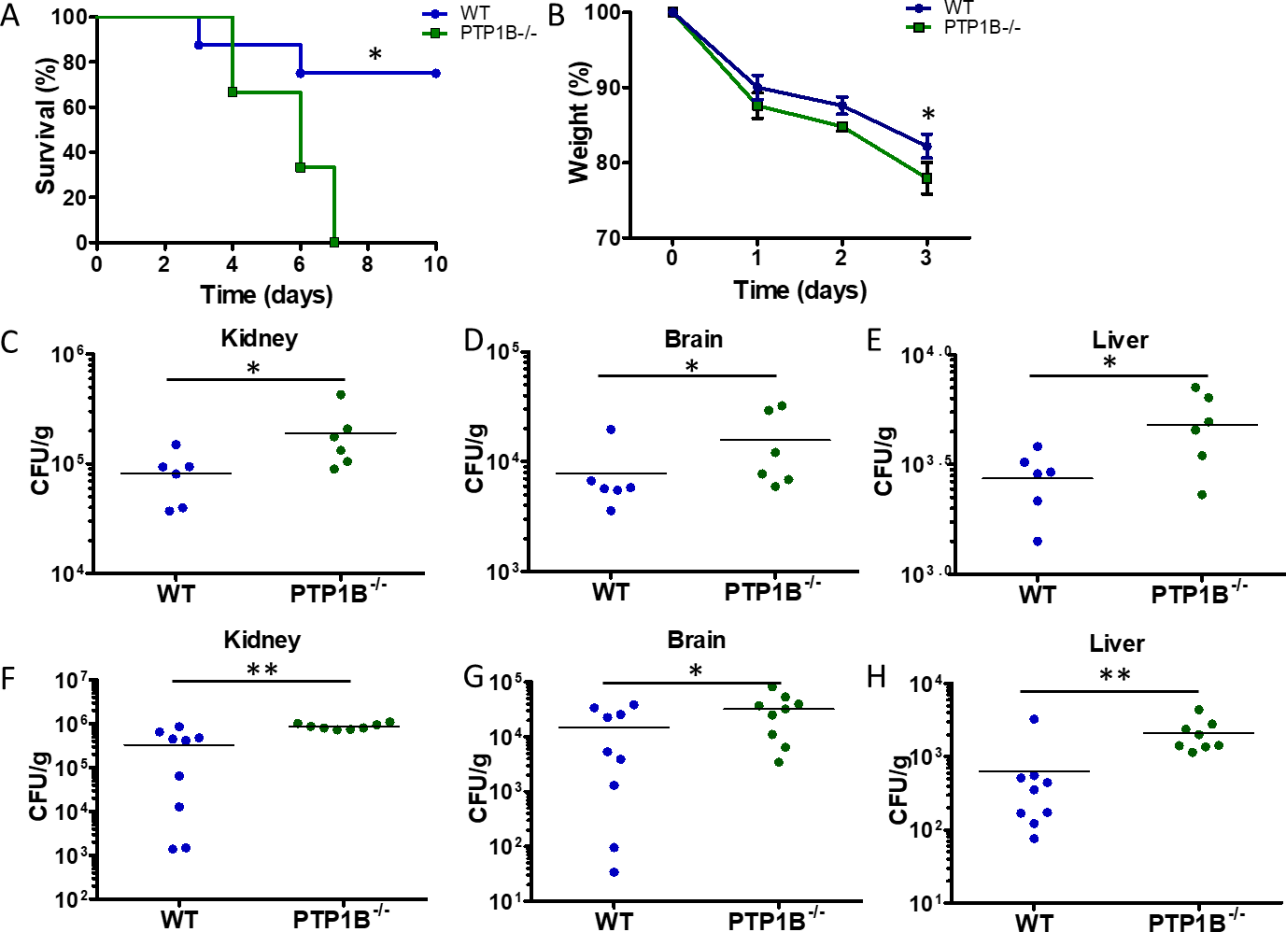
Myeloid cell PTP1B regulates antifungal immunity *in vivo*. (**A**) Survival curve of WT and LysM PTP1B^−/−^ mice after *C. albicans* challenge. WT and LysM PTP1B^−/−^ mice (nine mice per group) were infected intravenously with 2 × 10^5^ CFU of *C. albicans,* and percentage survival was calculated over the time period indicated. Data were pooled from two independent experiments. *P < 0.05, Mantel-Cox log-rank test. (**B**) WT and LysM PTP1B^−/−^ mice were infected with 2 × 10^5^ CFU of *C. albicans* and sacrificed 3 days post-infection; WT (*n* = 9) and LysM PTP1B^−/−^ (*n* = 8). Data are pooled from two independent experiments for each time point. Body weights were recorded for each mouse and expressed as percentage weight relative to total body weight prior to infection; one-way analysis of variance with Tukey multiple comparison test; *P < 0.05. (**C–H**) Organs were harvested, and the number of fungal colony-forming units per gram of tissue (CFU/g) was calculated for kidney, brain, and liver, either 24 h (**C–E**) or 3 days (**F–H**) post-infection; *P < 0.05, **P < 0.01 by Mann-Whitney U-test.

To assess the potential role of PTP1B in immune defense against fungal pathogens, we used our previously characterized mice with PTP1B knocked out in myeloid cells (LysM PTP1B^−/−^) and control littermate PTP1B^fl/fl^ mice, referred to hereafter as wild-type (WT) mice (11, 19). Mice were challenged intravenously with 2.5 × 10^5^ colony-forming units (CFU) with the standard laboratory strain of *C. albicans,* and survival was monitored over 10 days (Fig. 1A). This invasive candidiasis model recapitulates the severe sepsis that can occur in clinical cases, resulting in systemic hyperinflammation and renal failure (20). The majority of control WT mice showed resilience against *C. albicans* infection, with 78% surviving until the end of the study period (day 10). Conversely, LysM PTP1B^−/−^ mice were significantly more susceptible, with none surviving past 7 days post-infection, and the majority succumbing within 4 days. This increased susceptibility to *C. albicans* infection in LysM PTP1B^−/−^ mice was also observed when mice were challenged with a lower dose (1 × 10^5^ CFU/mouse) of *C. albicans* (Fig. S1A). Body weights were recorded prior to and up to 3 days post-infection, as an indicator of the health of the mice. Following infection, all mice decreased in weight; however, the weight loss was greater in the LysM PTP1B^−/−^ mice (mean percentage weight of initial body weight, day 3 post-infection; LysM PTP^−/−^ mice, 77.9%; WT 82.2%; *P* < 0.05), reflecting their increased susceptibility to infection (Fig. 1B; Fig. S1B). These differences in survival and weight loss illustrate the importance of myeloid PTP1B for host defense against systemic fungal infection. In line with this, LysM PTP1B^−/−^ mice demonstrated significantly greater fungal burdens in the kidneys, brains, and livers, 24 h (Fig. 1C through E) and 3 days (Fig. 1F through H) post-infection, as assessed by CFU counts.

**Fig 2:**
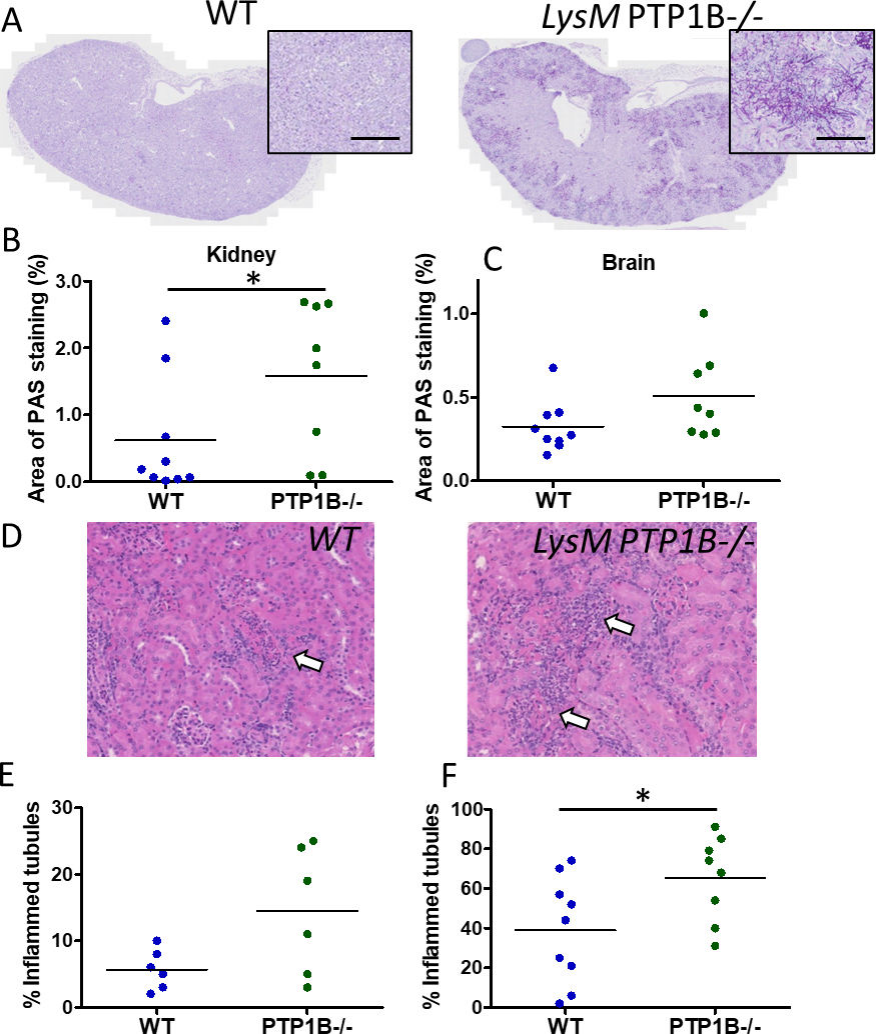
LysM PTP1B^−/−^ mice exhibit greater fungal lesions and inflammation than WT mice. (**A**) Kidney sections were stained with PAS to visualize *C. albicans* hyphae, and examples are shown for whole sections and magnified fungal lesions (scale bar shows 100 µm). The area of PAS staining, 3 days post-infection for kidney (**B**) and brain (**C**), as determined by ImageJ analysis, was calculated as a percentage of the total area of the section. *P < 0.01, Mann-Whitney U-test. (**D**) hematoxylin and eosin stain (H&E) stained 3 days post-infection kidney section from WT and PTP1B^−/−^ mice showing inflammation as indicated by white arrows. The percentage of inflamed tubules was calculated for WT and LysM PTP1B^−/−^ in H&E-stained kidney sections from mice culled 24 h (**E**) and 3 days (**F**) post-infection; *P < 0.05, by Student’s *t*-test with Welch’s correction.

Histological examination of periodic acid-Schiff (PAS)-stained tissue revealed a substantial presence of *C. albicans* hyphae in the kidneys of LysM PTP1B^−/−^ mice as compared to WT mice and an increase in fungal burdens, as analyzed by PAS staining in the kidneys and brains at 3 days post-infection (Fig. 2A through C). Lethality of systemically infected mice can result from inflammation-mediated damage to kidneys (20), reflecting human disease, thus the percentage of inflamed tubules (tubulointerstitial nephritis) in kidney sections from infected WT and LysM PTP1B^−/−^ mice was scored (Fig. 2D through F). Following 24 h of *C. albicans* infection, increased inflammation in the kidneys of LysM PTP1B^−/−^ mice was noted, indicative of increased leukocyte infiltration (Fig. 2E). By 3 days post-infection, the percentage of inflamed tubules was significantly greater (*P* = 0.04) in the kidneys of LysM PTP1B^−/−^ mice, with an average score of 64 ± 21% compared to 40 ± 28% in WT mice (Fig. 2F).

**Fig 3:**
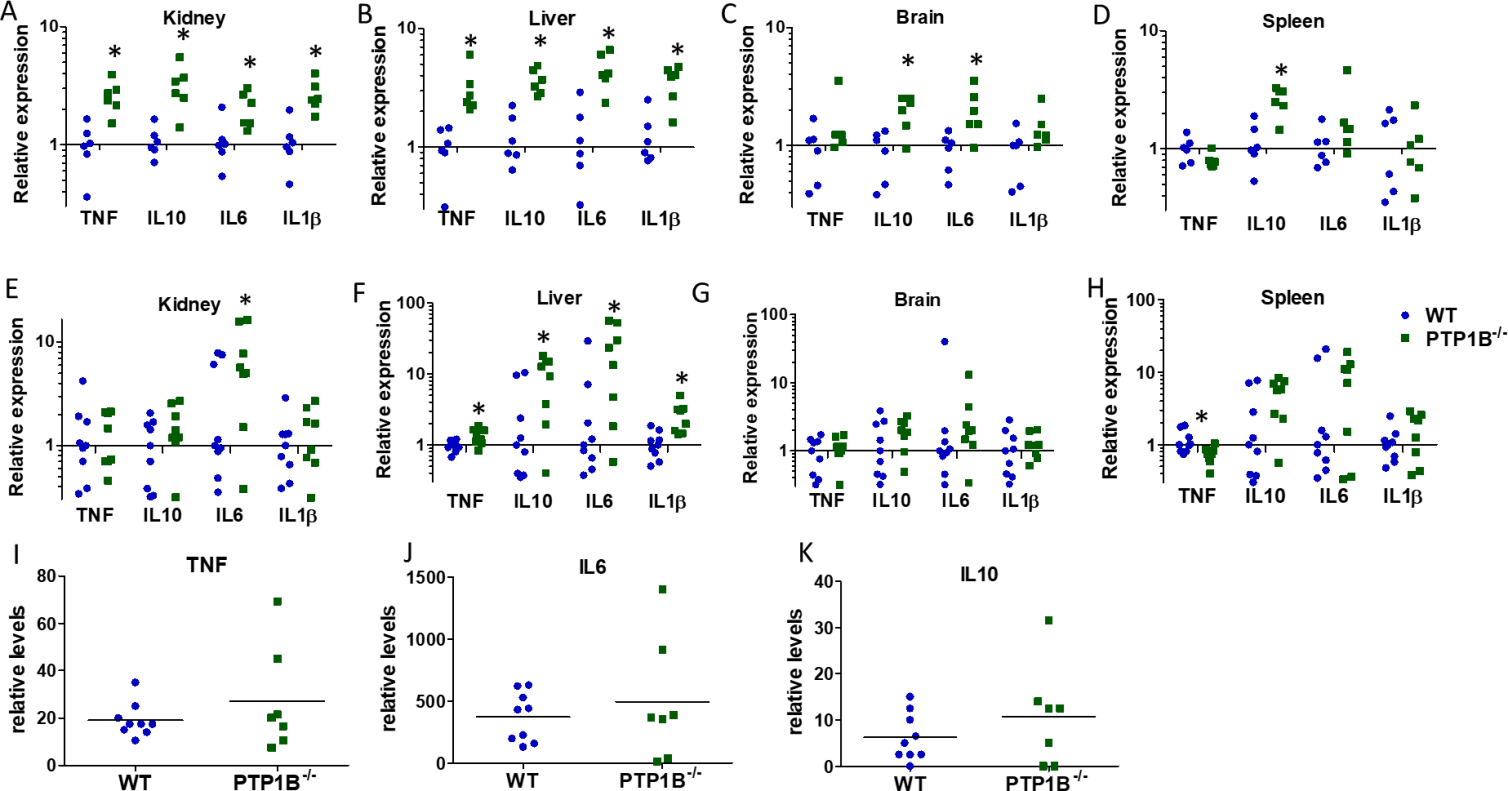
LysM PTP1B^−/−^ mice show increased tissue and systemic cytokine expression. WT and LysM PTP1B^−/−^ mice were infected with 2 × 10^5^ CFU of *C. albicans* and sacrificed 24 h post-infection (**A–D**) in two separate experiments (*n* = 3 in each experiment) or 3 days post-infection (**E–H**) in two separate experiments (*n* = 3, *n* = 6 for WT; *n* = 3, *n* = 5 for LysM PTP1B^−/−^). Fold change (2^-ΔΔCt^) in expression of *Tnf, Il6, Il1b,* and *Il10* mRNA in LysM PTP1B^−/−^ in kidney (**A, E**), liver (**B, F**), brain (**C, G**), and spleen (**D, H**) was calculated relative to median expression in WT. Bars represent mean ± SEM; *P < 0.05 represents significant difference between LysM PTP1B^−/−^ and WT. Serum was collected from mice 24 h post-infection, and levels of cytokines were analyzed by multiplex arrays. Serum levels of cytokines are expressed as relative levels from mean fluorescence intensity values minus background for TNF-alpha (**I**), IL-6 (**J**), and IL-10 (**K**). Values from individual experiments and mean are shown.

We sought to determine if the increase in histological inflammation and fungal burdens within organs at 24 h and 3 days post-infection in LysM PTP1B^−/−^ mice related to elevated expression of proinflammatory cytokines. These molecules are important for antifungal immunity, but their overproduction can result in hyperinflammatory responses, cellular damage, and fatal immunopathology during *C. albicans* infections (20). Tumor necrosis factor (TNF), interleukin-10 (IL-10), IL-6, and IL-1β are important players in the inflammatory response and were selected as candidates to analyze as they are known to be key cytokines showing early upregulated expression in organs during systemic *C. albicans* infection in the murine intravenous challenge model (21). *Tnf, Il6, Il1b,* and *Il10* mRNA levels were all significantly increased 24 h post-infection in the kidneys of LysM PTP1B^−/−^ mice compared to WT, which showed comparatively little upregulation at that time point (Fig. 3A), reflecting the lower fungal burdens observed in WT kidneys (Fig. 1C). Indeed, the levels of cytokines in kidney correlated significantly with that of fungal burdens, with kidneys of LysM PTP1B^−/−^ mice demonstrating higher levels of both (Fig. S2). Increases in *Tnf, Il6, Il1b,* and *Il10* mRNA levels were also detected in the liver and brain of LysM PTP1B^−/−^ mice compared to WT mice (Fig. 3B and C), in line with their elevated fungal burdens, and *Il10* mRNA levels were significantly increased in spleens 24 h post-infection (Fig. 3D). At 3 days post-infection, *Il6, Il1b,* and *Il10* mRNA levels were generally greater in the kidneys and livers for the majority of LysM PTP1B^−/−^ mice (Fig. 3E through H), again consistent with enhanced fungal burdens; significantly lower *Tnf* mRNA expression was, however, found in the spleen of LysM PTP1B^−/−^ mice. To define if the greater levels of inflammatory cytokines observed in organs of LysM PTP1B^−/−^ mice were also observed systemically, levels of serum cytokines were measured 24 h and 3 days post-infection. There was no significant difference in serum levels of TNF-alpha, IL-6, and IL-10 in LysM PTP1B^−/−^ mice (Fig. 3I through K) at 24 h post-infection, and at 3 days post-infection, serum cytokines were below the limits of detection of the multiplex assay. Thus, the degree of *C. albicans* infection correlates better with cytokines produced locally in the infected organ, as has been reported previously (20).

### Myeloid cell PTP1B negatively regulates *C. albicans*-induced chemokine expression and immune cell infiltration in infected organs

**Fig 4:**
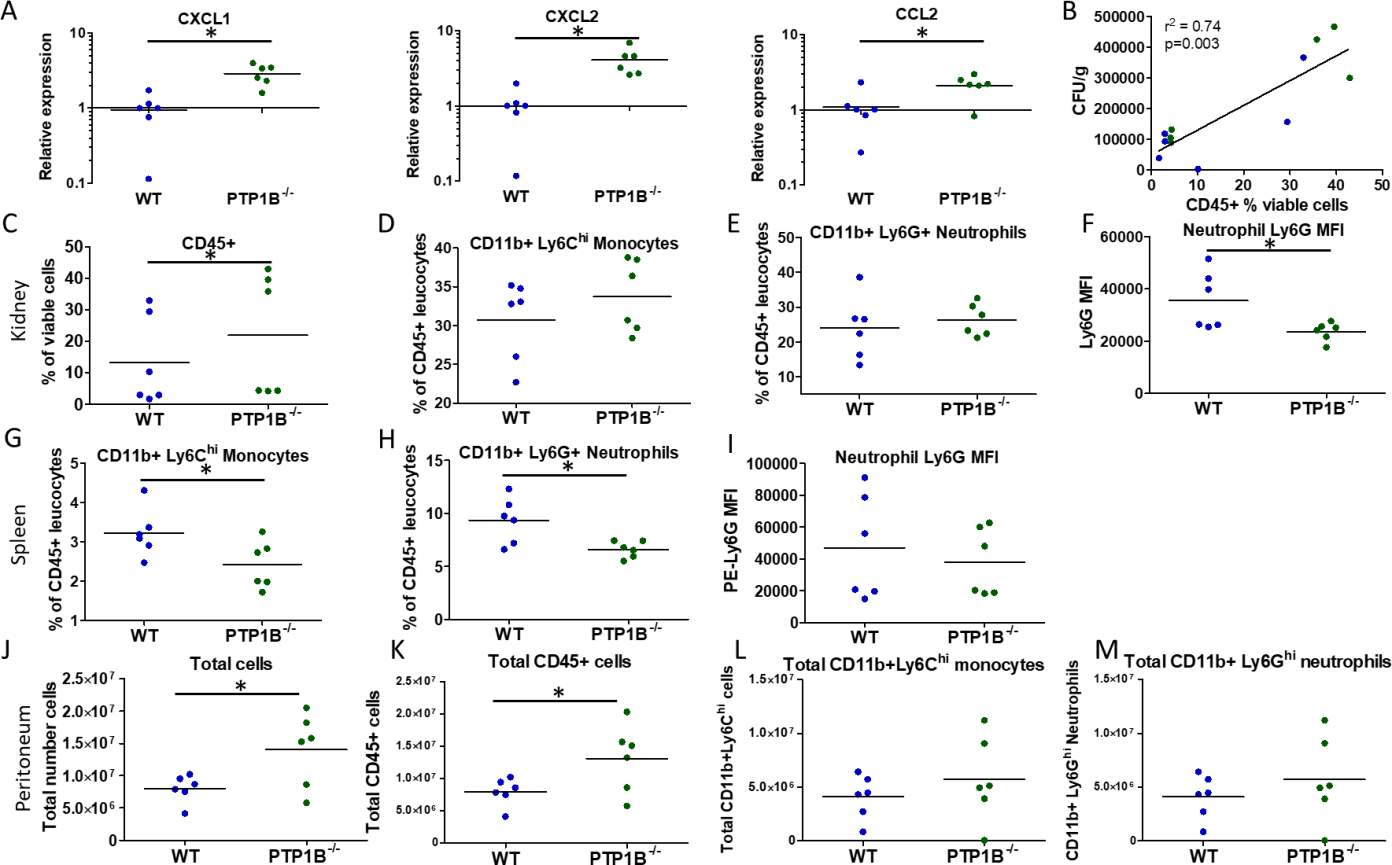
LysM PTP1B^−/−^ mice show increased chemokine expression and infiltrating immune cells in the infected kidney and peritoneum. Expression levels of chemokines *cxcl1, cxcl2,* and *ccl2*, as determined by real-time RT-qPCR, in WT and LysM PTP1B^−/−^ mice kidneys isolated 24 h post-infection with *C. albicans* (**A**). Results were normalized to the expression of the housekeeping genes and are presented as fold change relative to median expression in WT. *P < 0.05, Student's *t*-test. Correlations between proportion of CD45+ leukocytes out of total viable cells and fungal burdens (CFU/g) in kidney of WT (blue) and LysM PTP1B^−/−^ (green) mice at 24 h post systemic infection with *Candida albicans* (**B**). Pearson correlation and linear regression were used for statistical analysis. WT and LysM PTP1B^−/−^ mice were infected systemically with 2 × 10^5^ CFU of *C. albicans* and sacrificed 24 h post-infection in two separate experiments (*n* = 3 in each experiment). Flow cytometry was used to analyze the expression of immune cell markers in the kidney (**C–F**) and spleen (**G–I**). Total immune cells were those viable cells that were CD45+. Inflammatory monocytes were defined as CD45+ CD11b+ Ly6 C^hi^ (**D, G**), and neutrophils were defined as CD45+ CD11b+ Ly6G+ (**E, H**). Each cell population was expressed as a percentage of viable CD45+ leukocytes in the organ. CD45+ CD11b+ Ly6G+ neutrophils were analyzed for levels of Ly6G expression, shown by geometric mean fluorescence intensity (MFI) of PE-Ly6G antibody (**F, I**). *P < 0.05. WT and LysM PTP1B^−/−^ mice were infected intraperitoneally with *C. albicans* (1 × 10^6^ CFU) and peritoneal cells isolated 4 h post-infection and analyzed by flow cytometry. The total number of isolated peritoneal cells (**J**), CD45+ cells (**K**), inflammatory monocytes defined by CD45^+^ CD11b^+^ and Ly6C^hi^ (**L**), and neutrophils as defined by CD45^+^ CD11b^+^ and Ly6G^hi^ (**M**). *P < 0.05. Mann-Whitney U-test.

The increase in histological tubulointerstitial inflammation and enhanced expression of inflammatory cytokines in the primary target organ, the kidney, observed at 24 h post-infection, is suggestive of hyperimmune responses to *C. albicans* in LysM PTP1B^−/−^ mice. In line with this, we observed a significant increase in the expression of chemokine genes *CXCL1, CXCL2,* and *CCL2* in these kidneys (Fig. 4A). These chemokines promote recruitment of neutrophils and monocytes that can enhance inflammation. Quantification of the average proportion of CD45+ leukocytes out of total viable cells in infected kidneys (as an estimate of the proportion of infiltrating inflammatory cells) correlated significantly with level of fungal burden (Fig. 4B), and both were greater in LysM PTP1B^−/−^ mice. Proportions of viable CD45+ leukocytes and inflammatory monocytes showed an increased trend in kidneys of LysM PTP1B^−/−^ mice (Fig. 4C and D), in keeping with the significantly increased inflammation score (Fig. 2E and F) and chemokine expression. Proportions of kidney infiltrating PTP1B^−/−^ neutrophils were similar (Fig. 4E); however, they demonstrated significantly lower expression of Ly6G in LysM PTP1B^−/−^ mice compared to that observed in WT kidney, potentially indicating their less mature or less activated phenotype as previously described (19, 22, 23) (Fig. 4F, Fig. S3). Conversely, lower proportions of both neutrophils and inflammatory monocytes were found in the spleens of LysM PTP1B^−/−^ mice compared to WT (Fig. 4G and H), and splenic PTP1B^−/−^ neutrophils showed no significant change in Ly6G expression (Fig. 4I). To compare effects of myeloid PTP1B deficiency on localized rather than systemic infection, the infiltration of immune cells into the peritoneal cavity after *C. albicans* infection was determined. Following intraperitoneal *C. albicans* infection, the total number of cells and number of CD45+ cells isolated from the peritoneal cavity of LysM PTP1B^−/−^ mice 4 h post-infection was significantly higher than in WT mice, indicating, as with systemic infection, a greater localized organ inflammatory response in these animals (Fig. 4J and K). A moderate increase in the number of Ly6C monocytes and Ly6G neutrophils (Fig. 4L and M) was evident in LysM PTP1B^−/−^ mice, but differences did not reach statistical significance. Thus, PTP1B deficiency exacerbated inflammation, with enhanced chemokine expression and leukocyte recruitment in response to *C. albicans* infection.

### PTP1B deletion decreases neutrophil reactive oxygen species (ROS) production and killing capacity

**Fig 5:**
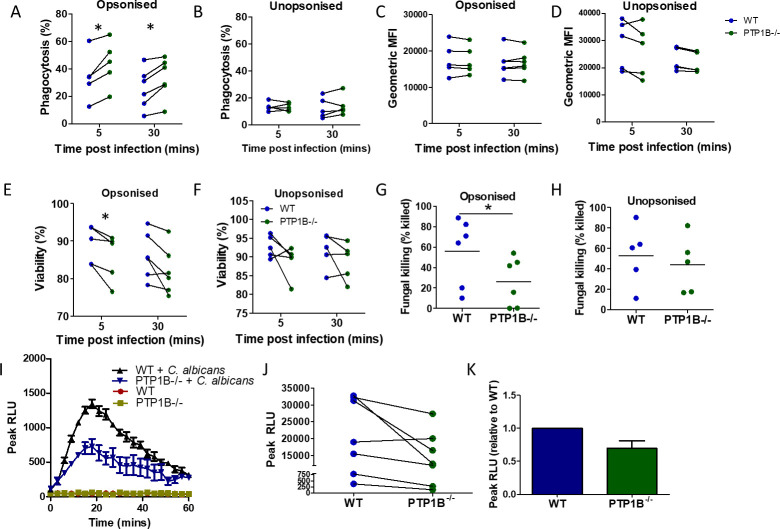
PTP1B controls the antifungal activity of neutrophils. Bone marrow-derived neutrophils isolated from age- and sex-matched LysM PTP1B^−/−^ and WT mice were infected with live serum-opsonized (**A, C, E, G**) or non-opsonized (**B, D, F, H**) *C. albicans* at a multiplicity of infection (MOI) of 10. Cells from two to three separate mice were pooled for each experiment (*n* = 5-6 separate experiments performed on separate days). Bone marrow-derived neutrophils were incubated with *C. albicans* for 5 or 30 min, and the phagocytic activity was measured by flow cytometry to determine the percentage fungal uptake (**A, B**) and the phagocytic index, determined as the geometric mean fluorescence intensity (MFI) (**C, D**). Isolated bone marrow-derived neutrophils were infected with live opsonized (**E**) or unopsonized (**F**) *C. albicans* at an MOI of 10 for 5 or 30 min, and viability was assessed using flow cytometry. Cells were gated for Ly6G (PE) expression, and cells negative for eFluor 780 (APC-Cy7) were defined as live. For A–F, paired lines represent the average values from LysM PTP1B^−/−^ and WT neutrophils from individual experiments. To determine killing efficiency, bone marrow-derived neutrophils were infected with live *C. albicans* yeast cells (MOI = 0.5) for 1 h and lysed. *C. albicans* at the same concentration, but without cells, was incubated for the same time as a control. The lysates were plated, and viable *C. albicans* cells were scored by counting the colonies formed on the plates (CFU) 24 h later (**G, H**). The percentage of killing = (colony number of control − colony number of neutrophil-treated)/colony number of control × 100. Bone marrow-derived neutrophils were infected with live *C. albicans* yeast cells (MOI = 10), and kinetics of ROS production was measured via a luminescence-based assay. Luminescence was measured as relative luminescence units (RLUs) to empty plate wells over 2 h (example shown in panel **I**, *n* = 1, mean ± SEM of quintuplicate technical replicates). Raw peak RLUs (**J**) and peak RLUs relative to WT (**K**) were recorded for each experiment (*n* = 7, mean ± SEM). *P < 0.05, two-way analysis of variance (A–F); Student’s *t*-test (G, H, K).

Given the increase in susceptibility to systemic fungal infection and greater inflammatory responses in LysM PTP1B^−/−^ mice, the functional differences in immune cells that could impact host defense were determined, firstly through evaluation of neutrophil phagocytic efficacy. There was inter-day variability in the percentage phagocytosis values between individual neutrophil preparations; therefore, paired data from WT and PTP1B^−/−^ neutrophil experiments performed on the same day was analyzed. A significantly greater percentage of LysM PTP1B^−/−^ bone marrow-derived neutrophils bound/internalized opsonized *C. albicans* (5 min post-infection, WT 34.2 ± 7.6 vs LysM PTP1B^−/−^ 44.3 ± 7.6; 30% upregulation; 30 min post-infection, WT 25.7 ± 6.0 vs LysM PTP1B^−/−^ 32.9 ± 7.8; 28% upregulation) (Fig. 5A). This increase in binding/internalization was not observed using unopsonized *C. albicans* (Fig. 5B), indicating that PTP1B deficiency specifically affects binding/uptake through opsonic receptors and their downstream signaling pathways that are known to play a key role in neutrophil clearance of fungi. Although the percentage of PTP1B^−/−^ neutrophils that bound/engulfed opsonized *C. albicans* was increased, no significant effect on the number of *C. albicans* associated on a per cell basis (Fig. 5C and D) was observed. This greater proportion of neutrophils engaging with *C. albicans* could potentially influence overall clearance. There was a general decrease in viability of PTP1B^−/−^ neutrophils compared to WT neutrophils for the majority of individual experiments following incubation with opsonized and unopsonized *C. albicans,* with opsonized *C. albicans* at 5 min post-infection showing a significant decrease (Fig. 5E and F). This decrease in viability of PTP1B^−/−^ neutrophils has the potential to impact their general ability to respond to fungal infection.

Next, the candidacidal activity of bone marrow-derived neutrophils was assessed against serum-opsonized and unopsonized *C. albicans,* where we evaluated their ability to decrease the number of fungi surviving following 1 h incubation. PTP1B deficiency in neutrophils led to impaired fungicidal activity for opsonized *C. albicans* with each neutrophil preparation (three mice per preparation, *n* = 6 per group) showing a reduced killing, which was on average 46.8% less than the killing ability of WT mice neutrophils (Fig. 5G), although this was not observed with non-opsonized *C. albicans* (three mice per preparation, *n* = 5 per group) (Fig. 5H). This decrease in killing could play a role in the enhanced susceptibility of LysM PTP1B^−/−^ mice to fungal infection, where *in vivo* most of the systemic *C. albicans* would be opsonized. A key killing mechanism of neutrophils to control *C. albicans* infection is through ROS production (24). We assessed ROS production induced by *C. albicans* stimulation in neutrophils from LysM PTP1B^−/−^ and WT mice over 2 h, and peak values over the course of the experiment were recorded (Fig. 5I through K). Peak luminescence values varied between individual experiments; however, ROS production was found to be lower in neutrophils from LysM PTP1B^−/−^ mice in six out of seven individual neutrophil preparations, with an average 30% decrease (Fig. 5J and K), in keeping with the lower neutrophil killing ability. Therefore, despite PTP1B-deficient neutrophils showing increased fungal binding/phagocytosis, they have lower ROS production and fail to kill opsonized *C. albicans* as effectively as WT cells, potentially relating to the increased organ fungal burdens observed *in vivo*.

### PTP1B deficiency in macrophages decreases their phagocytic capacity and killing ability but enhances inflammatory cytokine production

**Fig 6:**
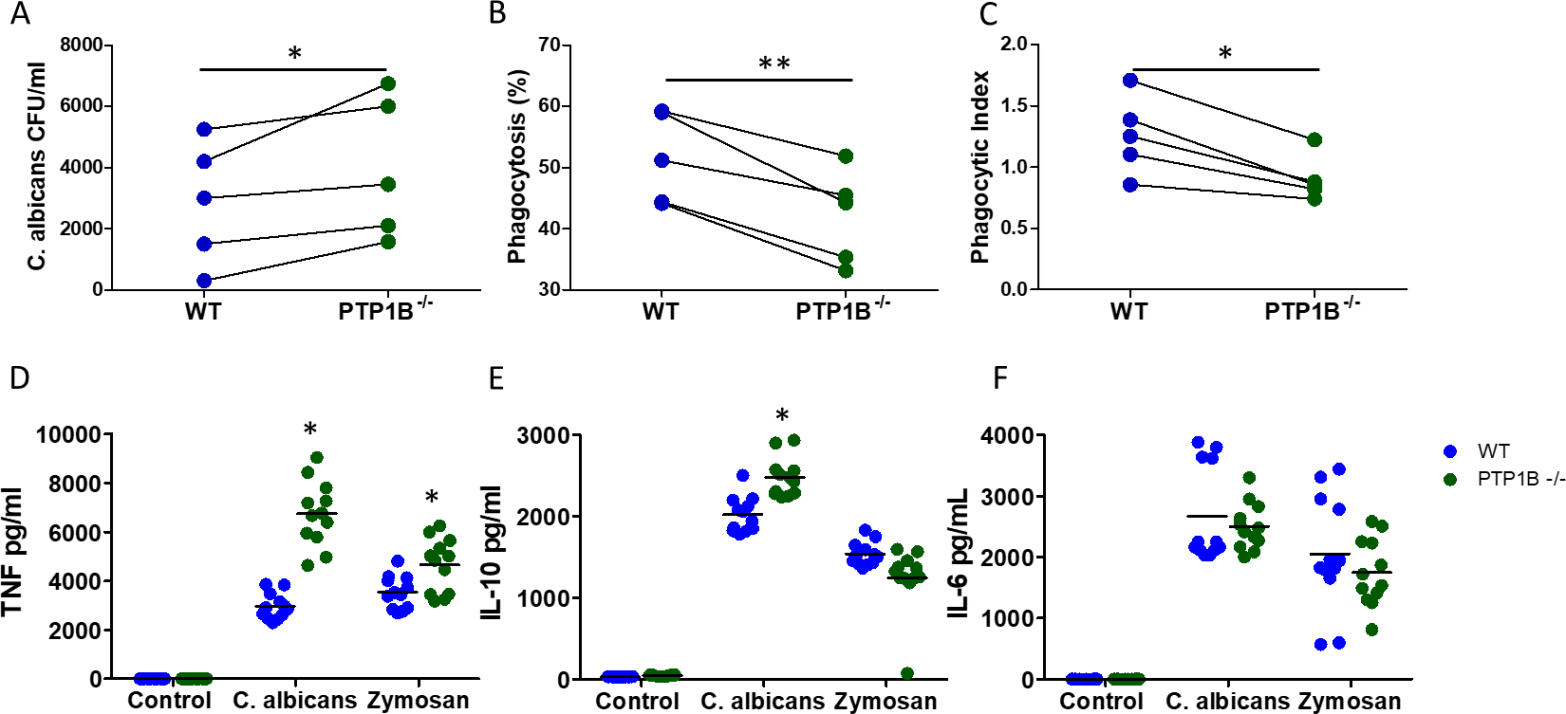
PTP1B regulates antifungal effector functions of macrophages. (**A**) BMDMs from age- and sex-matched mice were infected with live *C. albicans* yeast cells (multiplicity of infection (MOI) = 0.5) for 3 h, then lysed. Fungal CFUs were calculated per milliliter of lysis solution (*n* = 5) and used to define killing efficacy. BMDMs were infected with live fluorescein isothiocyanate-labeled *C. albicans* yeast cells (MOI = 1) for 3 h, and percentage uptake (**B**) and phagocytic index (**C**) of *C. albicans* were measured via microscopy (*n* = 5). Cytokine levels in supernatants of *C. albicans*-stimulated bone marrow-derived macrophages for TNF-alpha (**D**), IL-10 (**E**), and IL-6 (**F**) exposed to either heat-killed *C. albicans* or zymosan (100 µg/mL), as determined by Enzyme-linked immunosorbent assay (ELISA). Data are represented as mean ± SD (*n* = 12 individual BMDM preparations). Significant differences in levels of cytokine between WT and PTP1B^−/−^ BMDMs are shown; *P < 0.05, Student’s *t*-test.

Macrophages are also key immune responders in the prevention of *C. albicans* infections. To directly investigate the effect of PTP1B deficiency on the killing ability of macrophages, we co-cultured bone marrow-derived macrophages (BMDMs) with *C. albicans* and determined CFU of lysed cells 3 h post culture. As with PTP1B^−/−^ neutrophils, PTP1B^−/−^ BMDM showed significantly decreased fungicidal activity (increased CFU) when compared to WT BMDM, and this decrease was observed in each of the independent BMDM preparations (Fig. 6A). The phagocytic uptake and efficacy of uptake were next determined. BMDMs isolated from LysM PTP1B^−/−^ mice demonstrated competent uptake, indicating they were functionally capable of recognizing fungi; however, they were less able to phagocytose *C. albicans* than WT BMDMs (Fig. 6B). The number of *C. albicans* internalized by each BMDM (phagocytic index) was also decreased (Fig. 6C). Live cell imaging of uptake of *C. albicans* over a 3 h period (Fig. S4) indicated a comparable decrease in phagocytosis and phagocytic index over time in LysM PTP1B^−/−^ BMDM. The average rate of engulfment at the start of the phagocytosis process was 37.7 ± 2.1 min in LysM PTP1B^−/−^ BMDM, as compared to 23.2 ± 1.4 min in WT BMDM. In PTP1B^−/−^ BMDM, it took 95.2 ± 6.4 min for complete phagocytosis to occur as compared to 89.8 ± 9.2 min in WT BMDM (*n* = 2 individual BMDM preparations). Thus, the decrease in killing ability of PTP1B^−/−^ BMDM related to a delay in uptake kinetics.

BMDM-secreted cytokines drive inflammatory responses in immune cells. PTP1B has been shown to influence the magnitude of cytokine output upon activation by bacteria, viruses, and their components (12, 17). To determine whether PTP1B deficiency also influenced macrophage cytokine output following fungal activation, BMDMs were stimulated with *C. albicans*. Stimulation with *C. albicans* yeast cells resulted in low or undetectable levels of cytokines. Therefore, for these experiments, heat-killed *C. albicans* hyphae*,* known to induce greater cytokine production and release, or the fungal cell wall component, zymosan (100 µg/mL), was used to activate BMDMs and supernatants harvested. *C. albicans* hyphae-stimulated BMDMs from LysM PTP1B^−/−^ mice produced more TNF-alpha and IL-10 than BMDMs from WT mice (Fig. 6D and E). BMDMs from LysM PTP1B^−/−^ mice stimulated with zymosan, which signals through Dectin-1 and TLR2, also secreted more TNF-alpha but not IL-10, implying the increase in IL-10 in PTP1B^−/−^ cells is more likely to be driven through pathways distinct from Dectin-1 and TLR2. Stimulation with *C. albicans* hyphae or zymosan did not result in significant changes in levels of IL-6 in the supernatant of LysM PTP1B^−/−^ BMDMs compared to WT (Fig. 6F). To translate our findings, human blood monocyte-derived macrophages were isolated and treated with a pharmacological PTP1B inhibitor, MSI-1436, 0.7 µM, a dose that has previously been shown to inhibit PTP1B activity in other systems (6, 25). Human macrophages treated with MSI-1436 showed a similar increase in TNF-alpha but not IL-6 secretion after *C. albicans* hyphae or zymosan stimulation (Fig. S5). Levels of IL-1β and IL-12p40 in the culture supernatant were below the limit of detection following 3 h and 24 h stimulation with both mouse BMDM and human monocyte-derived macrophages. Taken together, our data show that PTP1B deficiency inhibits immune defense by macrophages through decreased phagocytosis and killing, in spite of the enhanced *C. albicans*-induced production of cytokines observed by the macrophages. Overall, this implies *C. albicans* is more effectively evading the innate immune system in PTP1B-deficient myeloid cells and *in vivo*, potentially increasing the risk of pathogen-induced systemic toxicity and the reduced survival of LysM PTP1B^−/−^ mice when challenged intravenously with *C. albicans*.

### Deletion of PTP1B alters the proteome of *C. albicans*-infected macrophages

**Fig 7:**
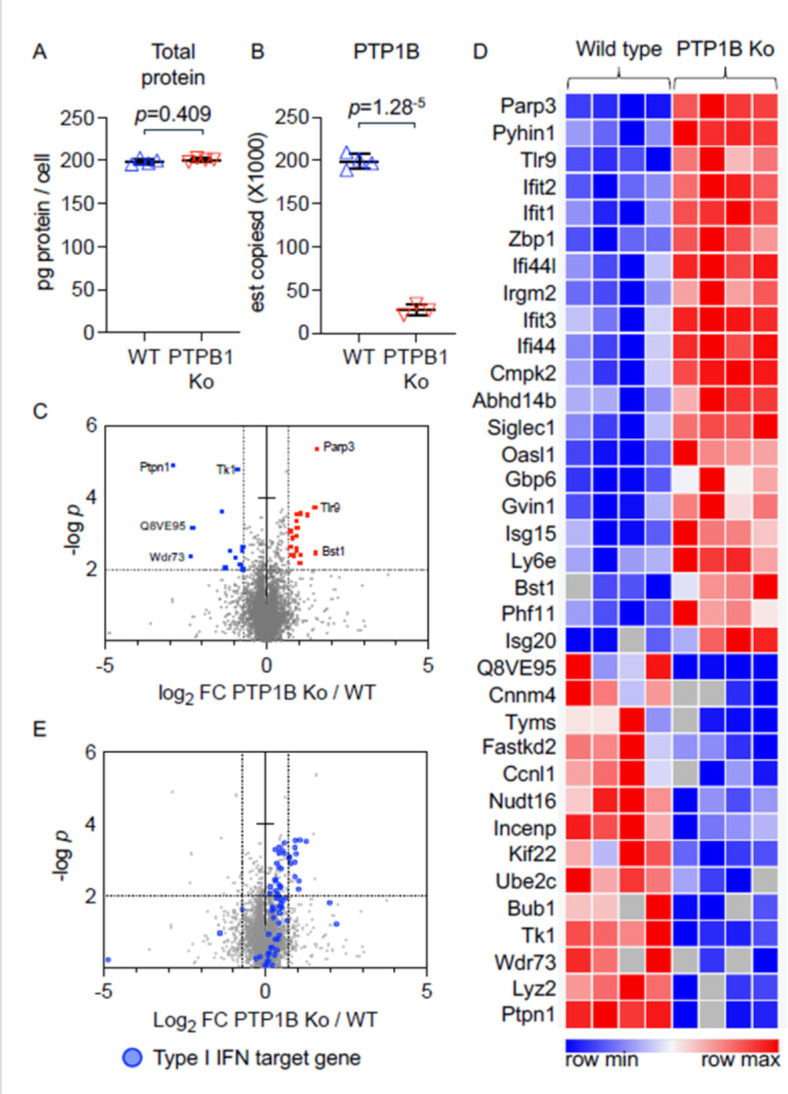
Deletion of PTP1B alters the proteome of *C. albicans*-stimulated macrophages. BMDMs from WT and LysM PTP1B^−/−^ mice were infected with live *C. albicans* (multiplicity of infection (MOI) = 4) for 8 h. Cells were then lysed and the proteomes analyzed using data-independent acquisition (DIA)-based mass spectrometry as described in Materials and Methods. Separate cultures from four mice per genotype were analyzed. (**A**) Total protein content (pg/cell) was estimated for the copy number data derived from the mass spectrometry results; *P*-value was determined by a two-tailed Student's *t*-test. (**B**) Estimated copy numbers for Ptpn1 from the wild-type and LysM PTP1B^−/−^ macrophages. (**C**) Volcano plot showing −log2 fold change in mean protein copy numbers per cell between *C. albicans*-infected WT and PTP1B^−/−^ BMDMs where cut-off thresholds are plotted as vertical lines. Upregulated (red) and downregulated (blue) proteins were defined as having a fold change more than two standard deviations away from the median and a *P*-value of less than 0.01 with cutoff threshold shown as horizontal line. (**D**) Heat map for the z-scores of the proteins classed as upregulated or downregulated in panel **C**. (**E**) Volcano plot showing the regulation of potential type I interferon-regulated proteins, highlighted in blue, differentially expressed between the wild-type and LysM PTP1B^−/−^ macrophages infected with *C. albicans*. Type I interferon-regulated proteins were defined based on a published transcription study in BMDMs (25).

To investigate potential mechanisms that may account for the defective fungicidal responses of PTP1B-deficient macrophages, label-free quantitative proteomics was utilized to identify differentially expressed proteins in WT and LysM PTP1B^−/−^ BMDMs that had been infected with live yeast *C. albicans*. The total cellular protein mass, estimated from the proteomic analysis, was approximately 200 pg/cell, with no major differences between WT and LysM PTP1B^−/−^ cells (Fig. 7A). As expected, PTP1B levels in the knockout BMDM were significantly reduced (Fig. 7B). There were 6,357 unique proteins identified (Table S1), and 35 proteins had significantly different expression levels (based on a log_2_ fold change greater than two standard deviations from the median value with significance *P* ≤ 0.01) between *C. albicans*-infected WT and PTP1B knockout BMDM, with 21 proteins showing an increase and 14 proteins with decreased expression (Fig. 7C and D). Gene Ontology (GO) enrichment did not reveal any significant hits for the downregulated proteins; however, upregulated proteins were associated with GO terms linked to responses to type I interferon and viral infection (Table 1). As there was an enrichment in proteins potentially controlled by type I interferon, the regulation of other interferon-stimulated genes (ISGs) in the proteomic data set was analyzed. The regulation of some ISGs can be cell type specific; therefore, a list of type I interferon-regulated genes was generated based on the transcriptomic studies reported by Mostafavi et al. (26). Of this list, 67 type I interferon-regulated proteins were identified in the proteomic data set, with these proteins showing a strong trend to be increased in the PTP1B^−/−^ macrophages relative to the WT cells (Fig. 7E, Table S2). This enhanced interferon signature in *C. albicans*-infected LysM PTP1B^−/−^ BMDM is consistent with the significant upregulation of interferon-induced chemokines observed in kidneys in our infection model (Fig. 4A) and with previous studies that have shown an association of increased interferon signaling responses with PTP1B knockout (10, 12, 14, 17, 18, 27). We also determined the expression of two interferon signature genes (*ifit2* and *stat1*) in the kidney, the primary target organ, of WT and LysM PTP1B^−/−^ mice following 24 h infection with *C. albicans*. There was a significant upregulation in expression of both these genes in LysM PTP1B^−/−^ murine kidneys, suggesting the enhanced interferon signature observed in LysM PTP1B^−/−^ macrophages is reflected in tissue and may be linked to the greater inflammatory response and leukocyte infiltration (Fig. S6A).

**TABLE 1:** GO term enrichment of proteins upregulated in LysM PTP1B^−/−^ macrophages^*a*^

Enrichment FDR	*n* Genes	Pathway genes	Fold enrichment	Pathway	Biological process	Gene name
1.37E−04	3	21	102.88	GO:0035457	Cellular response to interferon-alpha	*Ifit1 Ifit2 Ifit3*
9.92E−04	3	35	54.47	GO:0035455	Response to interferon-alpha	*Ifit1 Ifit2 Ifit3*
9.52E−06	5	57	49.78	GO:0035458	Cellular response to interferon-beta	*Ifi209 Irgm2 Ifit1 Ifit3 Gbp6*
1.21E−05	5	66	41.71	GO:0035456	Response to interferon-beta	*Ifi209 Irgm2 Ifit1 Ifit3 Gbp6*
1.85E−05	7	299	15.00	GO:0051607	Defense response to virus	*Ifit1 Isg20 Oasl1 Tlr9 Ifit3 Zbp1 Isg15*
1.85E−05	7	300	15.00	GO:0140546	Defense response to symbiont	*Ifit1 Isg20 Oasl1 Tlr9 Ifit3 Zbp1 Isg15*
1.21E−05	8	384	12.86	GO:0009615	Response to virus	*Ifit1 Isg20 Oasl1 Tlr9 Ifit3 Zbp1 Isg15 Ifit2*
5.12E−05	8	774	9.50	GO:0009617	Response to bacterium	*Gbp6 Cmpk2 Isg15 Tlr9 Ifi44 Ifit1 Irgm2 Ifit3*
9.52E−06	10	804	8.87	GO:0045087	Innate immune response	*Zbp1 Gbp6 Tlr9 Irgm2 Ifit1 Isg15 Isg20 Oasl1 Ifi209 Ifit3*
5.12E−05	9	851	7.69	GO:0071345	Cellular response to cytokine stimulus	*Zbp1 Ifi209 Irgm2 Gbp6 Ifit1 Ifit3 Isg15 Oasl1 Ifit2*
1.27E−04	9	961	6.78	GO:0034097	Response to cytokine	*Zbp1 Ifi209 Irgm2 Gbp6 Ifit1 Ifit3 Isg15 Oasl1 Ifit2*

^*a*^ Proteins upregulated in the knockout were defined as having a fold change greater than the median fold change value plus two standard deviations from the proteomic data set and a *P*-value of less than 0.01. Enrichment was carried out against a background comprising all the proteins identified in the proteomic data set for GO terms for biological processes that contained between 10 and 1,000 members. GO terms passing an FDR cutoff of 0.001 are shown.

As well as increasing the expression of STAT1, type I interferons also promote STAT1 phosphorylation. Given that PTP1B is a phosphatase, the functional changes relating to PTP1B knockout in macrophages would involve post-translational modifications of protein as well as changes in the level of protein expression (6). Accordingly, we compared phosphorylation levels of STAT1 in WT and LysMPTP1B^−/−^ BMDM either activated with *C. albicans* yeast or left unstimulated. *C. albicans* did not enhance phosphorylation of STAT1. In both the absence (basal levels) and presence of *C. albicans,* macrophages from LysMPTP1B^−/−^ mice show heightened phosphorylation of STAT1 that can drive the enhanced type I interferon (IFN) signature we observed with proteomic analysis (Fig. S6B and C). Moreover, we have previously shown elevated STAT1 and STAT3 phosphorylation in lipopolysaccharide (LPS)-activated BMDMs from LysM PTP1B^−/−^ mice (11).

### PTP1B deficiency in macrophages modifies macrophage metabolism and impacts their viability following *C. albicans* infection

**Fig 8:**
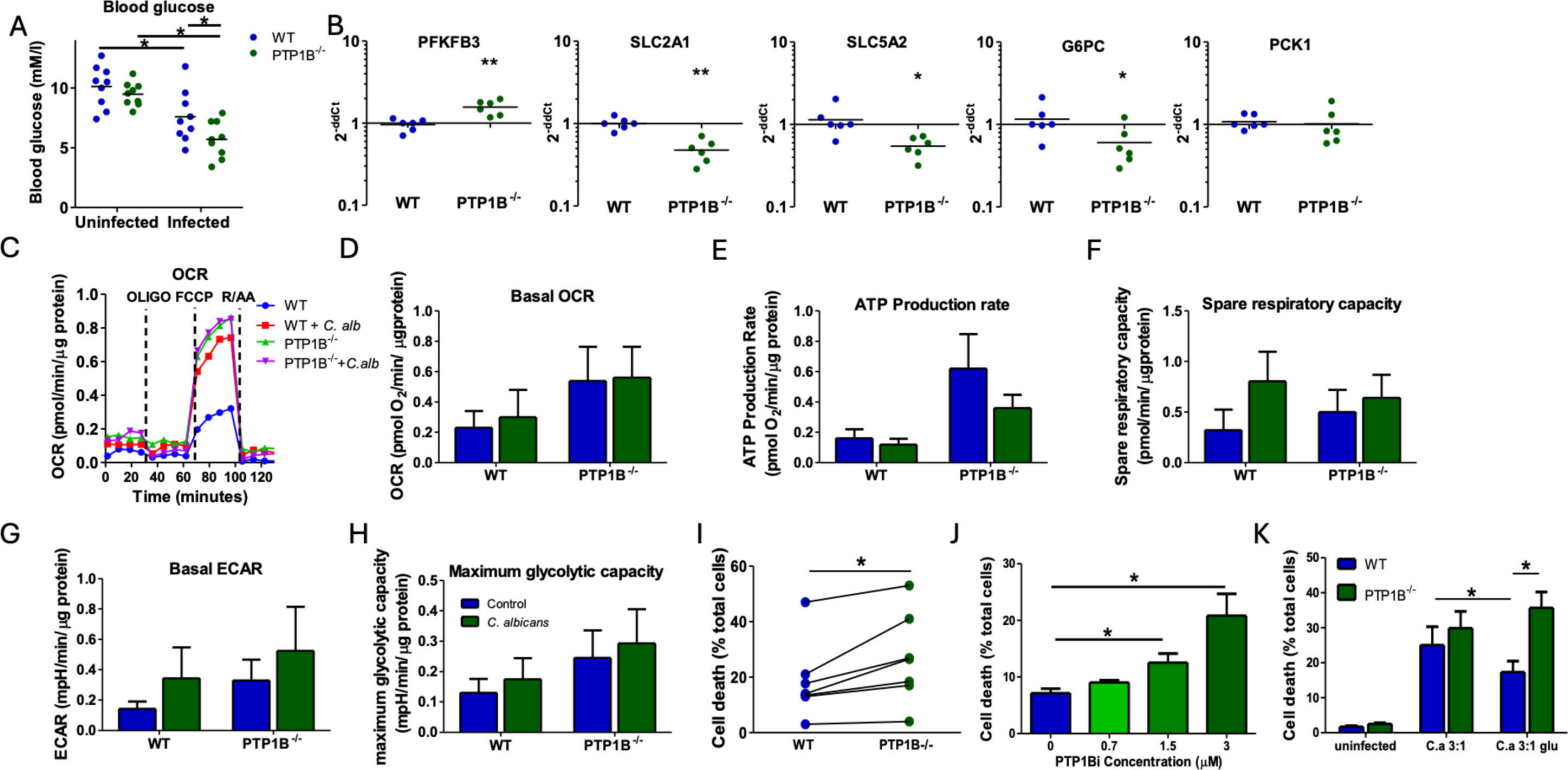
Deletion of PTP1B alters the metabolic profile of BMDM that consequently decreases their viability and increases susceptibility to infection. (**A**) Blood glucose levels in uninfected and 24 h post *C. albicans*-infected (2 × 10^5^ yeast cells/mouse) WT and LysM PTP1B^−/−^ mice. (**B**) Quantitative PCR analysis of metabolic gene expression in kidneys of 24 h post *C. albicans*-infected WT or LysM PTP1B^−/−^ mice for glycolytic enzyme Pfkfb3, glucose importers *SLC2A1* (GLUT1) and *SLC5A2* (SGLT2), and gluconeogenic genes *G6PC* and *PCK1*. Results were normalized to the expression of the housekeeping genes and are presented as fold change relative to median value of WT BMDM. (**C**) A Cell Mito Stress Test was performed using a Seahorse XFe24 Analyzer and oxygen consumption rates (OCRs) measured with representative example of WT and LysM PTP1B^−/−^ BMDM with and without heat-killed *C. albicans* activation shown. (**D**) Basal OCR, (**E**) ATP production by mitochondria, (**F**) spare respiratory capacity, (**G**) basal ECAR, and (**H**) maximum glycolytic capacity of macrophages were measured with or without heat-killed *C. albicans* in WT and LysM PTP1B^−/−^ BMDM and normalized with cell numbers. Data are shown as mean ± SEM, n = 4–5 separate BMDM preparations. (**I**) Cell death as a percentage of total cells in WT and PTP1B^−/−^ BMDM infected with live *C. albicans* MOI 1:1. (**J**) Cell death as a percentage of total human monocyte-derived macrophage following treatment with 0, 0.7, 1.5, or 3.0 µM PTP1B inhibitor MSI1436. (**K**) Cell death as a percentage of total cells in WT and PTP1B^−/−^ BMDM infected without or with live *C. albicans* multiplicity of infection (MOI) 1:3 with or without addition of glucose 20 mM. Shown are mean values and SEM for five to six separate BMDM preparations, or three to four individual human monocyte preparations from each group performed in triplicate. * = *P* < 0.05, ** = *P* < 0.01; analysis of variance (ANOVA) (**A**, **B**, **I**) or ANOVA with Tukey’s multiple comparisons *post hoc* test (**D**–**H**, **J**, **K**). For graph **K**, uninfected WT and LysM PTP1B^−/−^ BMDM were significantly different from WT and LysM PTP1B^−/−^ BMDM without and with glucose; however, significant differences are not marked for clarity.

Infection causes a metabolic stress in host tissues, organs, and immune cells, as well as systemically, and this can result in energy deficits and tissue damage (28, 29). In line with this, infection with *C. albicans* results in a decrease in circulating blood glucose levels in WT mice (Fig. 8A). PTP1B is a crucial regulator of metabolic pathways and glucose homeostasis (6), and this infection-induced hypoglycemia was even more pronounced in LysM PTP1B^−/−^ mice. In LysM PTP1B^−/−^-infected mouse kidneys as the primary target organ in systemic *C. albicans* infection, we observed a significant increase in the expression of the glycolysis regulator *PFKFB3* compared to WT mice, yet expression of *SLC2A1* (GLUT1), and *SLC5A2*, the main glucose transporter in kidneys, was decreased (Fig. 8B). LysM PTP1B^−/−^ mice also show significantly less expression of *glucose 6 phosphatase* (*G6PC*) in infected kidney that could result in decreased gluconeogenesis, while expression levels of the gluconeogenic gene *phosphoenolpyruvate carboxykinase 1* (*Pck1*) were similar (Fig. 8B).

Immune cells modify their metabolism and increase glycolytic pathways to drive killing of pathogens, and the killing ability of macrophages from LysM PTP1B^−/−^ mice was blunted. To determine whether myeloid PTP1B deficiency could also reconfigure metabolism individually in macrophages in a manner that alters effective host defense, we used the Seahorse extracellular flux analyzer to analyze potential changes. We used heat-killed *C. albicans* yeast to focus results on infected macrophages and avoid changes in metabolism resulting from live *C. albicans* metabolic adaptation. Culture of WT macrophages with *C. albicans* tended to increase respiration rates over that of uninfected macrophages, as evident by a generalized increase in oxygen consumption rate (OCR) as a measure of oxidative phosphorylation and extracellular acidification rates (ECAR) as a measure of glycolysis (Fig. 7C through H). These results are consistent with previous studies in live *C. albicans*-infected macrophages and monocytes (30, 31) and indicate that WT macrophages support the ability of the cells to respond to increasing energy demands on infection. Knockout of PTP1B resulted in a trend of higher basal metabolism over that of WT BMDM as evidenced by a greater basal OCR, mitochondrial ATP production rate, basal ECAR, and maximal glycolytic capacity (Fig. 7C through H). Following *C. albicans* infection, the increase in OCR, and spare respiratory capacity (maximum use of mitochondrial capacity) seen in WT macrophages was not as evident in LysM PTP1B^−/−^ macrophages, potentially relating to their higher basal levels. Thus, WT and LysM PTP1B^−/−^ macrophages have differential capacities to alter metabolism upon infection. A similar alteration in metabolism was observed in human blood-derived macrophages treated with pharmacological PTP1B inhibitor MSI-1436, 1.5 µM (Fig. S7A and B), where a significant increase in basal ECAR and glycolytic capacity over that of macrophages without drug treatment was observed with and without infection. These results imply that with *C. albicans,* human or murine macrophages with inhibited or deficient PTP1B, respectively, rely more heavily on glucose as a substrate to meet increased energy demands.

Glucose depletion is a key mechanism of *C. albicans*-induced death of macrophages (30). As LysM PTP1B^−/−^ macrophages show greater dependence on glycolysis following infection, we hypothesized that macrophage death would be enhanced in infected LysM PTP1B^−/−^ macrophages where *C. albicans* competes with activated macrophages for the enhanced glucose needed to exert antifungal defense. We therefore determined the viability of *C. albicans*-infected LysM PTP1B^−/−^ macrophages after 6 h incubation with *C. albicans* (multiplicity of infection [MOI] = 1). There was a significant increase in cell death in *C. albicans*-infected macrophages with PTP1B deficiency (Fig. 8I; Fig. S7C), and this was recapitulated in infected human monocyte-derived macrophages treated with a pharmacological PTP1B inhibitor, MSI-1436, at concentrations of 1.5 and 3 µM; *P* < 0.05, (Fig. 8J). The greater death of LysM PTP1B^−/−^ *C. albicans*-activated macrophages could potentially relate to their higher metabolic rate and greater dependence on glucose following infection. Cell death following *C. albicans* infection can be partially rescued by addition of glucose (30). We repeated experiments using an MOI of 3 to stimulate greater infection-induced death, with and without addition of glucose 3 h post-infection, and analyzed cell death 3 h later. At an MOI of 3, the increase in cell death observed in *C. albicans*-infected LysM PTP1B^−/−^ macrophages did not reach significance (Fig. 8K). Addition of glucose significantly decreased the degree of cell death in infected WT macrophages; however, addition of glucose failed to rescue cell death in LysM PTP1B^−/−^ macrophages, reflecting the altered metabolic dynamics on infection in these cells. Taken together, the decrease in LysM PTP1B^−/−^ macrophage defenses against *C. albicans* could relate to metabolic alterations that are no longer sufficient to maintain functional efficacy.

## DISCUSSION

PTP1B plays a key role in orchestrating several cell signaling cascades, including those of oncogenic, metabolic, and inflammatory relevance (6, 8). Here, we have uncovered the significance of myeloid cell PTP1B in regulating a broad range of antifungal innate immune responses to *C. albicans*. Consequently, mice deficient in myeloid cell PTP1B are more susceptible to systemic infection, with increased mortality, body weight loss, and organ fungal burdens that enhance pathogen-induced systemic toxicity. Kidneys, as the main target organ in systemic *C. albicans* infection, exhibit substantially increased inflammation, chemokine and cytokine expression, and leukocyte infiltration in LysM PTP1B^−/−^ mice, confirming an overexuberant inflammatory phenotype and correlating with their enhanced fungal burdens and immunopathology. In spite of an enhanced inflammatory response *in vivo*, neutrophils from LysM PTP1B^−/−^ mice show dampened *C. albicans*-induced ROS production and killing ability as compared to WT neutrophils. PTP1B-deficient BMDMs show an enhanced interferon-responsive signature yet have impaired functional phagocytosis and killing responses against *C. albicans*, potentially relating to rewired metabolism and accelerated glucose starvation-induced death, effects also observed in human macrophages treated with a pharmacological PTP1B inhibitor. Therefore, PTP1B-regulated signaling pathways play a key role in modulating several functional properties of myeloid cells that control fungal infection, and collectively, these new data provide a mechanistic explanation for the greater vulnerability to fungal infection in LysM PTP1B^−/−^ mice.

Increased susceptibility to *C. albicans* infection can result from enhanced organ fungal burdens due to deficiencies in immune cell function and lack of clearance or overwhelming inflammation leading to tissue destruction, both of which manifest in infected LysM PTP1B^−/−^ mice. In general, compared to WT mice, more immune cells infiltrate the kidney and peritoneum of LysM PTP1B^−/−^ mice following *C. albicans* infection, and this increased infiltration links to the greater expression of kidney chemokines. Enhanced immune cell infiltration and chemokine expression have been reported in other disease models of global PTP1B^−/−^ mice (9, 16, 32). In line with our myeloid-specific PTP1B deficiency, global PTP1B deficiency exacerbated allergic inflammation, with heightened leukocyte recruitment, early expression of proinflammatory mediators, chemokines and leukocyte chemokine receptors, and more rapid recruitment of effector cells from bone marrow and the peripheral circulation (32). PTP1B^−/−^ mice infected with respiratory syncytial virus also exhibited exaggerated immune cell infiltration (16), and in a separate study, ear edema induced by tetradecanoylphorbol acetate was more severe in PTP1B^−/−^ mice due to higher neutrophil accumulation and leukocyte trafficking (9). Thus, PTP1B commonly functions to limit the recruitment of leukocytes during inflammatory and allergic responses, and its deficiency enhances immune cell infiltration.

We clearly demonstrate the knockout of PTP1B specifically in myeloid cells significantly worsened susceptibility to systemic fungal infection. This contrasts with the defensive role of PTP1B knockout observed in other types of infection. For example, studies using global PTP1B knockout mice reported protection against *Pseudomonas aeruginosa* infection (12, 18) despite a common increase in neutrophil recruitment and proinflammatory cytokine expression in both studies. We have also shown previously that myeloid cell PTP1B deficiency in mice protects against endotoxemia and high-fat diet-induced inflammation (11). Yet, PTP1B^−/−^ mice infected with respiratory syncytial virus exhibit exaggerated immune cell infiltration, damaged epithelial cell barriers, cytokine production, and aggravated overall lung injury (16). The multifunctional activity and function of myeloid PTP1B are therefore dependent on the context and role of immune cells in individual diseases, and pathogen-specific differences and the signaling pathways they initiate. Likewise, mice lacking expression of another class of phosphatase, suppressor of TCR signaling 1 (Sts-1)^−/−^ mice, are resistant to disseminated candidiasis and hyperinflammation, thereby showing the specificity of responses by the different phosphatases during infection (33).

Neutrophils isolated from LysM PTP1B^−/−^ mice show several defects that prevent their full activity in fungal defense that could partially account for a reduction in survival *in vivo*. Firstly, neutrophils infiltrating infected kidneys have a less mature phenotype (lower Ly6G expression) as compared to WT mice. Secondly, while PTP1B deficiency in cultured neutrophils enhances fungal clearance via phagocytosis, it leads to reduced ROS production, diminished killing capacity, and decreased cell viability. The reduced killing ability of PTP1B-deficient neutrophils was only significantly different from that of WT mice when fungi were opsonized, suggesting a key defect occurred via signaling through uptake receptors for opsonized particles. Yue et al. (18) also found phagocytosis of *Pseudomonas aeruginosa* was enhanced in PTP1B-deficient neutrophils, concomitant with increased expression of the opsonic receptor Fcgr1 and upregulated interferon regulatory pathways and cytokine production. We did not analyze the levels of Fcgr1 in neutrophils, but enhanced expression could explain the specificity of why only opsonized *C. albicans* showed a significant increase in uptake. Given that *in vivo,* most of the systemic *C. albicans* will be opsonized by circulating complement and antibodies, these *in vivo* changes in uptake with opsonized *C. albican*s have biological relevance. Our *ex vivo* cultured PTP1B^−/−^ macrophages similarly exhibited reduced fungal killing capacity; however, unlike neutrophils, they showed decreased phagocytic efficacy against *C. albicans*, likely due to the modulation of an alternative uptake pathway (9–12).

Proteomic analysis established that the key proteins modified in *C. albicans-*infected PTP1B-deficient macrophages relate to responses to type 1 interferons. PTP1B is a negative regulator of type I IFN signaling in several cell types, including macrophages, and PTP1B silencing enhances STAT1 activation that in turn augments IFN secretion (18, 34, 35). It remains to be determined whether the upregulation of interferon signature proteins observed in our infected PTP1B-deficient macrophages is linked to enhanced STAT1 activation observed in this study. IFN type I signaling pathways have been implicated in host defense against *C. albicans* infection, although this is intricate and their role has been debated (30, 35). Augmented type I IFN signaling also mediates significant hyperinflammation and fatal immunopathology during murine systemic *C. albicans* infections (35). The augmented IFN upregulates IFN-induced chemokines, including CCL2, and promotes recruitment and activation of inflammatory monocytes and neutrophils to infected organs. This causes the lethal effects of *C. albicans*-induced sepsis (35), events significantly enhanced in our LysM PTP1B^−/−^-infected mice. IFN-I-regulated IFIT proteins were significantly upregulated in our PTP1B-deficient macrophages, and these are known to inhibit activation of NADPH oxidase and ROS production and to upregulate genes promoting macrophage apoptosis and susceptibility to *C. albicans* infection (36, 37). We also observed enhanced expression of *ifit2 and stat1* as well as *cxcl1*, *cxcl2,* and *ccl2* in infected kidneys of our LysM PTP1B^−/−^ mice. These interferon signature genes are associated with inflammatory responses and tissue damage in invasive *C. albicans* infection, suggesting they would contribute to the overall pathology and increased susceptibility to fungal infection in our model. Interferon-related pathways also regulate host glucose metabolism and macrophage metabolic dysfunction during bacterial and viral challenges (38, 39). LysM PTP1B^−/−^-infected mice show lower blood glucose levels than those of infected WT mice, along with differences in the expression levels of metabolic enzymes. Whether these changes in host glucose metabolism are linked to altered interferon signaling pathways in LysM PTP1B^−/−^ mice warrants more detailed investigation.

Following *C. albicans* infection, macrophages reshape their metabolism to meet the energy-demanding process of host defense. *C. albicans* also switches to aerobic glycolysis, resulting in competition for available glucose, which can lead to local glucose depletion and trigger macrophage death due to starvation (30). PTP1B is a well-recognized regulator of metabolism, and our studies show that PTP1B knockout or inhibition enhances overall respiration and their dependence on glucose following infection. Under resting conditions, the upregulation in basal glucose consumption by PTP1B-deficient macrophages can be tolerated, but following the stresses of *C. albicans* infection, excess glucose usage and its depletion leads to functional insufficiency and augments cell death. A similar phenomenon has been observed in breast cancer cells with PTP1B deficiency, where both mitochondrial and non-mitochondrial respiration are elevated, and under hypoxic stress, these cells exhibit decreased functional activity and accelerated death (9, 40). Metformin induces comparable metabolic rewiring and accelerates macrophage death during *C. albicans* infection by shutting down the mitochondrial respiratory chain and driving faster glucose consumption (30).

In summary, this study has determined that the loss of PTP1B in myeloid cells significantly impairs the ability of mice to combat fungal infection, highlighting new roles for PTP1B in myeloid cell biology and providing key insights into antifungal immune defenses. As the number of patients at risk for candidiasis increases, it is crucial to gain a deeper understanding of the defense mechanisms against *C. albicans* to prevent and treat infection. We demonstrate the critical role of myeloid cell PTP1B in preventing excessive inflammatory and type I interferon responses and immune cell infiltration into tissues, as well as in regulating the ability of neutrophils and macrophages to kill *C. albicans* by affecting ROS production, phagocytosis, metabolism, and cell viability. Our findings have important clinical implications for how antifungal therapies may influence PTP1B levels and downstream signaling activity. Furthermore, as new PTP1B inhibitors advance in clinical trials, it will be important to assess how they impact susceptibility to infection and how different pathogenic clinical isolates impact this. Future studies will determine whether the increased sensitivity to *C. albicans* we observed in murine studies and human macrophages is fully recapitulated in patients treated with potential clinical PTP1B inhibitors, or if individuals with PTP1B defects, such as SNPs, are at higher risk of invasive candidiasis.

## MATERIALS AND METHODS

### *C. albicans* culture and opsonization

The standard reference *C. albicans* strain*,* SC5314, was grown at 30°C with shaking in yeast extract peptone dextrose (YPD) medium (1% yeast extract, 2% mycological peptone, 2% D-glucose). *C. albicans* was washed and resuspended at the required concentration in phosphate buffered saline (PBS). To opsonize *C. albicans*, 5 × 10^7^ *C. albicans* were resuspended in 200 µL mouse serum and incubated at 37°C for 15 min with frequent mixing. Opsonized *C. albicans* was washed three times with PBS prior to use.

### *In vivo* studies

PTP1B^fl/fl^ mice (referred to as WT mice) and mice expressing Cre under the LysM promoter (LysM PTP1B^−/−^ mice) were generated as described previously (11, 41) and genotyped using PCR with the following primers: PTP1B^fl^ (forward 5′-TGCTCACTC ACCCTGCTACAA-3′ and reverse 5′-GAAATGGCTCACTCCTACTGG-3′) (41). For all mouse experiments, 8- to 12-week-old mice were housed in ventilated cages (three to four per cage), maintained at 22°C–24°C on a 12 h light-dark cycle, and supplied with food and water *ad libitum*. For systemic infection survival studies, age-matched female mice were infected intravenously with 2.5 × 10^5^ or 1 × 10^5^ CFU of *C. albicans* (SC5314) per mouse and monitored daily for survival for up to 10 or 21 days, respectively, then euthanized. Animals were monitored daily for clinical symptoms and weight loss. Mice displaying pre-specified criteria for distress (inability to feed or drink, labored breathing, ruffled and/or matted fur, decreased activity, hunched posture, shivering, or weight loss <30%) were euthanized by cervical dislocation or high CO_2_, and tissues were collected post-euthanasia. To prepare tissue for histological analysis, fungal burden quantification, blood and serum collection, and molecular biology analysis, mice were infected intravenously with 2.5 × 10^5^ CFU of *C. albicans* (SC5314) and euthanized 24 h or 3 days post-infection via cervical dislocation, and organs were harvested immediately. For peritoneal infection, *C. albicans* SC5314 was intraperitoneally injected into 8- to 12-week-old age-matched mice (1 × 10^6^ yeast cells per mouse). Mice were monitored for clinical symptoms and euthanized 4 h post-infection via CO_2_ inhalation, and peritoneal cells were harvested immediately using ice-cold PBS containing 3% fetal bovine serum (FBS) injected into the peritoneum and collected after massaging the peritoneum to dislodge the cells.

### Fungal burden analysis

Organs were harvested, weighed, and homogenized, then serial dilutions were prepared. Neat or diluted homogenate (100 µL) was plated onto YPD agar (Sigma-Aldrich, MO, USA) and incubated at 30°C for 24 h. Colonies were counted and expressed as CFU per gram of tissue.

### Histology

Following harvest, organs were fixed in formalin for 24–48 h, washed, then embedded in paraffin wax blocks by the NHS Grampian Pathology Department. Sections of organs (4 µm thick) were stained with H&E and/or PAS stain according to standard protocols. Levels of inflammation in the kidney were scored by an NHS Grampian pathologist (Dr. Moira Davie) and assessed blind to infection status and genotype. For each section, 100 tubules were analyzed for the presence or absence of inflammatory cells, and inflammation was expressed as a percentage of affected tubules. To determine fungal burdens via PAS staining, slides were scanned (Axio Scan.Z1, Zeiss), and ImageJ analysis was used to calculate the stained area as a percentage of the total area of the section.

### Flow cytometric analysis of organ and peritoneal cells

Kidney tissue was suspended in dissociation cocktail (1.6 mg/mL collagenase and 200 µg/mL DNase I in Roswell Park Memorial Institute [RPMI] medium) and dissociated mechanically by gentleMACS Octo Dissociator for 30 min at 37°C (Miltenyi Biotec, Bergisch Gladbach, Germany). Kidney, spleen, bone marrow, or peritoneal cells were passed through a 100 µm cell strainer before being centrifuged at 400 × *g* for 10 min at 4°C, and cells were resuspended for analysis. All subsequent steps occurred at 4°C. Red blood cells (RBCs) were lysed using RBC lysis buffer (BioLegend, CA, USA) for 5 min. Cells were washed in fluorescence-activated cell sorting (FACS) buffer (Hanks' balanced salt solution [HBSS], 5% fetal calf serum [FCS], 5 mM EDTA) and incubated with 1:300 eFluor 780 fixable viability dye (Invitrogen, CA, USA) for 30 min at 4°C in the dark. Samples were washed in FACS buffer and fixed in 4% paraformaldehyde for 10 min, then stored overnight. Samples were incubated with 10 µg/mL anti-mouse CD16/CD32 Fc block (BD Biosciences, Oxford, UK) for 5 min. For characterization of immune cells in kidney and spleen, the antibody panel included CD45 Qdot 655 (Clone HI30), CD11b PerCp-Cy5.5 (Clone M1/70), Ly6G PE (Clone 1A8), CD64 AF647 (Clone X54-5/7.1), and Ly6C fluorescein isothiocyanate (FITC) (Clone AL-21), all from BD Biosciences and used at 0.67 µg/mL, apart from Ly6C added at 1.67 µg/mL. Cells were incubated with antibodies for 1 h in the dark before washing and resuspending in FACS buffer. Analysis was performed on the BD LSR Fortessa using FACSDiva software (BD Biosciences, Oxford, UK), followed by further analysis in FlowJo, LLC for Windows, version 10.4.2 (TreeStar Inc., Ashland, OR, USA). Gates for individual markers of interest were set based on fluorescence-minus-one or isotype controls, respectively, with acceptable background/unspecific staining signals of ≤1% of the parent. The gating strategy was performed as described in Fig. S8. Neutrophils were defined as CD45+ CD11b+ Ly6G+ cells and inflammatory monocytes were defined as CD45+ CD11b+ Ly6 C^hi^ cells. *t*-distributed stochastic neighbor embedding (t-SNE) analyses were performed using FlowJo v.10.6.1. t-SNE was calculated based on expression of CD45, CD11b, Ly6C, and Ly6G clusters.

### Bone marrow-derived monocyte/macrophage and neutrophil isolation

Uninfected mice were euthanized via carbon dioxide inhalation followed by cervical dislocation. Bone marrow was flushed from femurs and tibiae of mice using RPMI medium containing 10% fetal calf serum and passed through a 100 µm cell strainer (42). All procedures were performed at 4°C unless otherwise stated. RBC lysis buffer (BioLegend, CA, USA) was added to cells before washing and resuspending in PBS. Histopaque 1119 was overlaid with Histopaque 1077 (Sigma-Aldrich, MO, USA), which in turn was overlaid with bone marrow. Neutrophils (polymorphonuclear) and mononuclear cells were isolated following centrifugation at 700 × *g*, at 25°C for 40 min (no brake). Mononuclear cells were resuspended in supplemented Dulbecco's modified Eagle medium (DMEM) containing 10% heat-inactivated fetal calf serum, penicillin/streptomycin, and 1% Glutamax (Gibco Life Technologies), and neutrophils were used immediately. For BMDMs, mononuclear cells were plated on 10 cm Petri dishes in the above medium containing 20% L929 cell culture supernatant and allowed to differentiate BMDMs in a 37°C incubator for 6–8 days, supplementing half the medium every 3 days. For preparation of BMDMs used for proteomic analysis, recovered bone marrow cells were cultured in 2-mercaptoethanol (50 µM), non-essential amino acids 1× (Gibco), 10% heat-inactivated fetal calf serum, 1% sodium pyruvate (Lonza), HEPES (10 mM) (Lonza), and 20% L929 cell culture supernatant. Monocytes were allowed to differentiate into BMDMs in a 37°C incubator for 6–8 days on 10 cm petri dishes, then macrophages were reseeded onto tissue culture plastic in fresh media and left overnight before use.

### Human monocyte-derived macrophages

Blood samples were collected into EDTA vacutainers (BIO-Greiner), and peripheral blood mononuclear cells (PBMCs) were isolated from whole blood using density centrifugation. Blood was diluted 1:2 with HBSS (Lonza) before being overlaid with 15 mL Lymphoprep (Stem Cell Technologies) and centrifuged at 1,800 rpm for 40 min. PBMCs were counted and resuspended in 80 µL PEA buffer (PBS, 2.5 mmoL/EDTA [Gibco] and 0.5% human serum albumin [Alburex 5, 50 g/L] and 20 µL CD14^+^ microbeads [Miltenyi Biotec]) per 10^7^ cells added. PBMCs were incubated with CD14^+^ microbeads for 15 min at 4°C. Cells were washed with 2 mL PEA buffer per 10^7^ cells and centrifuged at 300 × *g* for 10 min. LS columns (Miltenyi Biotec) were washed with 3 mL PEA buffer before the bead and cell suspension was added. Columns were then washed three times before being removed from the magnet, and CD14^+^ cells were eluted with 5 mL PEA buffer. Human monocytes were cultured in RPMI (Lonza) supplemented with 5% heat-inactivated autologous serum and differentiated into monocyte-derived macrophages after 7 days.

### RNA isolation, reverse transcription, and qPCR

Organs or cells were manually homogenized in 1 mL TRIzol (Invitrogen, CA, USA) prior to centrifugation at 12,000 × *g* for 10 min at 4°C. The supernatant was collected and incubated for 5 min at room temperature. Chloroform (200 µL) (Thermo Fisher Scientific, MA, USA) was added, and samples were shaken for 15 seconds then incubated for 3 min at room temperature (RT). Samples were centrifuged (at 12,000 × *g* for 15 min at 4°C), and the clear upper phase was collected. Qiagen RNeasy Mini Kit (Qiagen, Hilden, Germany) was used to extract the RNA according to the manufacturer’s protocol, including addition of DNase. Oligo (dT) primer (Promega, WI, USA) and dNTP mix (Bioline, TN, USA) were added to 2 µg RNA in RNase-free water. Samples were incubated at 65°C for 5 min, then on ice for 3 min. SuperScript II (Invitrogen, CA, USA) master mix was prepared according to the manufacturer’s protocol and incubated with samples for 2 min at 42°C. SuperScript II reverse transcriptase was added, and reverse transcription was performed on a thermocycler via the following program: 42°C for 50 min, 70°C for 15 min, and finally >5 min at 4°C. The total volume was adjusted to 100 µL with RNase-free water, and cDNA samples were stored at −70°C prior to use. The Roche primer and probe system was used for all qPCR analysis (Roche, Basel, Switzerland; Table 2). LightCycler 480 Probes Master, primers, probes, and PCR-grade water were mixed according to the manufacturer’s protocol. The master mix (7.5 µL) was added to each well in a 384-well plate on ice, and 2.5 µL cDNA was added. No reverse transcriptase and no template controls were used as negative controls. Plates were analyzed on a Roche 480 LightCycler qPCR machine. For the expression of *ifit2* and *stat1*, available TaqMan probes (Applied Biosystems) were used for qPCR reactions as follows: *stat1* (Mm01257286_m1), *ifit2* (Mm00492606_m1), *actb* (Mm02619580_g1), *nono* (Mm00834875_g1). Samples were run on a StepOne Plus Real-Time PCR system (Applied Biosystems) in technical and biological replicates. Relative abundance was calculated by comparing expression to *Nono* or *ActB* RNA levels.

**TABLE 2:** Primers and probes (Roche) used for qPCR analysis

Gene	Primer left	Primer right	Probe
*NoNo*	CCCCACCAATACCTGCAA	TTCAGGTCAATAGTCAAGCCTTC	40
*ActB*	AAGGCCAACCGTGAAAAGAT	GTGGTACGACCAGAGGCATAC	56
*Hprt1*	TCCTCCTCAGACCGCTTTT	CCTGGTTCATCATCGCTAATC	95
*tnfα*	CTGTAGCCCACGTCGTAGC	TTGAGATCCATGCCGTTG	25
*il-10*	CAGAGCCACATGCTCCTAGA	TGTCCAGCTGGTCCTTTGTT	41
*il-6*	GCTACCAAACTGGATATAATCAGGA	CCAGGTAGCTATGGTACTCCAGAA	6
*il-1β*	AGTTGACGGACCCCAAAAG	AGCTGGATGCTCTCATCAGG	38
*cxcl1*	AGACTCCAGCCACACTCCAA	TGACAGCGCAGCTCATTG	83
*cxcl2*	AATCATCCAAAAGATACTGAACAAAG	TTCTCTTTGGTTCTTCCGTTG	56
*ccl2*	CATCCACGTGTTGGCTCA	GATCATCTTGCTGGTGAATGAGT	62
*Slc2a*	GGATCCCAGCAGCAAGAAG	CCAGTGTTATAGCCGAACTGC	76
*slc5a2*	GCTGGATTTGAGTGGAATGC	CACACCAGCGGTCAGATAC	68
*g6pc*	TCTGTCCCGGATCTACCTTG	GAAAGTTTCAGCCACAGCAA	19
*pck1*	ATGTGTGGGCGATGACATT	AACCCGTTTTCTGGGTTGAT	105
*pfkfb3*	AACAGCTTTGAGGAGCGTGT	CCGGGAGCTCTTCATGTTT	27

### *C. albicans* killing by macrophages and neutrophils

BMDMs were harvested from initial cultures using 0.05% trypsin/EDTA (Thermo Fisher, Scientific) and reseeded into a 12-well plate at a density of 5 × 10^4^ cells per well. The following day, cells were incubated with *C. albicans* (MOI = 0.5) for 1.5 h before washing with HBSS to remove extracellular fungal cells, then incubated for a further 1.5 h. BMDMs were lysed in 1.5 mL 0.1% Triton X-100 at 37°C for 10 min. Neutrophils isolated from bone marrow were seeded at 1 × 10^5^ cells per well and incubated with *C. albicans* (MOI = 0.5) for 1 h. Neutrophils were lysed in 1.5 mL 0.1% Triton X-100 for 10 min at 37°C. *C. albicans* at the same concentration but without cells was incubated for the same time in the same buffers as a control. The lysates were plated onto YPD agar plates, and viable *C. albicans* cells were scored by counting the colonies (CFU) 24 h later. The percentage of killing was calculated using the following formula: percentage killing = (colony number of control − colony number of neutrophil/macrophage-treated)/colony number of control × 100.

### Phagocytosis assays

#### BMDM

*C. albicans* was incubated with 1 mg/mL FITC (Sigma-Aldrich, MO, USA) in 50 mM carbonate/bicarbonate buffer (Na_2_CO_3_ + NaHCO_3_ in water, pH 9.6) for 10 min and washed in PBS. BMDMs were reseeded in a 12-well plate (5 × 10^4^ cells per well), and FITC-labeled *C. albicans* was added the following day (MOI = 1). After 3 h of incubation, BMDMs were washed in PBS and fixed in 4% paraformaldehyde for 10 min before analysis via fluorescence microscopy (Zeiss Observer Z1). Percentage uptake of *C. albicans* was determined by calculating the number of BMDMs containing one or more *C. albicans* cells as a percentage of total BMDMs. Phagocytic index was defined as the mean number of *C. albicans* cells per BMDM (43). For live video microscopy, BMDMs were seeded in μ-Slide 8-well chambers at a cell density of 5 × 10^4^ cells in 120 µL RPMI prior to the day of infection. BMDM samples were infected with FITC-labeled *C. albicans* at MOI 1 in 240 µL of CO_2_-independent RPMI (supplemented with 5% FCS and 1% penicillin/streptomycin) immediately prior to imaging. In some experiments assessing cell viability, 0.6 mM DRAQ7 (Abcam) for tracking macrophage membrane permeabilization was added. Live video microscopy was conducted using an UltraVIEW VoX 3D spinning disk confocal microscope at 20× objective, and the environment was set to 37°C and 5% CO_2_. Images were taken every 2 min over a 3 h period with Volocity 6.3 software (PerkinElmer) at different well locations containing approximately 40 macrophages. Videos were analyzed using the viewer software, Volocity 6.5.1 (Quorum Technology). The time points at the beginning and at the end of full yeast cell engulfment were recorded. The phagocytic index was calculated from the number of engulfed *C. albicans* over time. Data were obtained in triplicate from two separate experiments by analyzing at least 50 macrophages.

#### Neutrophils

FITC-labeled *C. albicans* were added to neutrophils (2 × 10^5^ cells per well) at MOI = 10 and incubated for 5–30 min at 37°C, and phagocytic activity was determined by flow cytometry. Neutrophils were incubated for 5 min with rat anti-mouse CD16/CD32 mAb (BD Biosciences) then labeled for 1 h at 4°C with anti-Ly6G antibody (BD Biosciences) conjugated to phycoerythrin (PE). Cells were centrifuged at 300 *g* for 5 min at 4°C, washed three times, and suspended in ice-cold FACS washing buffer for flow cytometry. Amine-reactive compensation beads (Invitrogen) with eFluor 780 viability dye and CompBeads (BD Biosciences) were used as compensation controls. The samples and controls were analyzed using the BD LSR Fortessa flow cytometer, and the acquired data were further analyzed using FlowJo v.10.6.1. The extent of phagocytosis/binding was expressed as the mean fluorescence intensity (MFI) of neutrophils and relates to the average number of FITC-labeled *C. albicans* phagocytosed or bound/associated with neutrophils.

### Cytokine quantification

#### ELISAs

Macrophages were seeded at 2 × 10^5^ per well/500 µL in 24-well plates and cultured overnight at 37°C in 5% CO_2_. After overnight incubation, *C. albicans* (MOI 1:50), heat-killed for 40 min at 65°C, or zymosan 100 µg/mL was added followed by a 3 h incubation at 37°C in 5% CO_2_. Supernatants from BMDMs (untreated or *Candida*-treated) were tested for cytokine production by ELISA using matched paired antibodies specific for TNF-alpha (BD Biosciences TNFα (Mono/Mono) ELISA Kit (555268), IL-10 (R&D SYSTEMS Mouse IL-10 DuoSet ELISA kit (DY417-05), and IL-6 (eBioscience Mouse IL-6 ELISA Ready-SET-Go! Kit (88-7064-22) and methodology in line with the manufacturer’s instructions. Absorbance changes were measured using a LABsystems Multiscan MS plate reader. Cytokine concentrations were calculated from standard curves generated by the Genesis software based on a quadratic regression curve.

#### Mouse Cytokine Luminex assay

To determine levels of IL-1, IL-6, IL-10, and TNF-alpha in mouse serum, a Mouse XL Cytokine Luminex Performance Premixed Kit (R&D Systems) was used as per the manufacturer’s instructions. Sample quantification was performed using a Luminex analyzer. Levels were quantified based on the MFI of standards and expressed as relative levels compared to these standard values.

### ROS production assays

Luminol (100 µM) (Sigma-Aldrich, MO, USA) was added to a 96-well microtiter plate containing 2.5 × 10^5^ neutrophils per well. *C. albicans* (50 µL, MOI = 10) was added, or phorbol-12-myristate-13-acetate (PMA) (400 ng/mL) was used as a positive control. Kinetic luminescence reads were performed on a Synergy HT reader (BioTek, VT, USA) every 3 min for 2 h. Each measurement was performed in quintuplicate. Relative luminescence units (RLUs) were calculated relative to empty plate wells, and the maximum (peak) RLU for each well during the course of the assay was recorded.

### Western blot

WT and LysM-PTP1B BMDMs or neutrophils were incubated with heat-killed *C. albicans* for defined time points. Samples were washed twice in PBS, radioimmunoprecipitation assay (RIPA) buffer (Thermo Fisher Scientific, Loughborough, UK) with Halt 100× protease and phosphatase inhibitor (Thermo Fisher Scientific) was added, and samples were pipetted vigorously. Lysates were incubated on ice for 30 min before centrifuging (17,000 × *g*, 15 min) and isolating supernatant. Total protein concentration was calculated using Pierce BCA assay (Thermo Fisher Scientific). NuPAGE LDS 4× sample buffer and β-mercaptoethanol were added to the lysate, and samples were incubated at 95°C for 5 min before loading into NuPAGE 4 to 12% Bis-Tris mini protein gels (Thermo Fisher Scientific, MA, USA). Gels were run in 1× NuPAGE MES SDS running buffer (Thermo Fisher Scientific) at 200V. Protein was transferred onto a 0.45 µm polyvinylidene difluoride (PVDF) membrane (GE Healthcare, IL, USA) via a wet transfer system in 1× NuPAGE transfer buffer (Thermo Fisher Scientific) with 10% methanol (for one gel). Protein transfer occurred at 30V for 1 h. Membranes were blocked in blocking buffer (5% milk in 0.05% PBS-Tween 20) for a minimum of 1 h. Primary antibody in blocking buffer was added for 1 h before washing three times in 0.05% PBS-Tween for 10 min. This step was repeated for horseradish peroxidase (HRP)-conjugated secondary antibody. Membranes were incubated in Pierce ECL western blotting substrate for 1 min prior to analysis on the iBright imager (Thermo Fisher Scientific).

### Proteomics

BMDMs (1 × 10^6^) were infected with live SC5314 *C. albicans* (MOI 4:1) for 8 h and lysed using 400 µL/1 × 10^6^ cells with 5% SDS (20% SDS Sigma Aldrich, 05030), 10 mm Tris(2-carboxyethyl)phosphine (TCEP) (0.5 M TCEP; Thermo Fisher Scientific, 77720), 50 mM triethylammonium bicarbonate (TEAB) (1 M TEAB; Thermo Fisher Scientific, 90114), and HiPerSolv Water for high-performance liquid chromatography (HPLC) (VWR, 83650.320). Samples from independent macrophage preparations from four mice per genotype were analyzed. Proteomic sample preparation was carried out using S-Trap purification using S-Strap: Rapid Universal MS Sample Prep (Co2-mini, Protifi) and trypsin digestion using Trypsin Gold (Promega, V5280) as described (44, 45). For each sample, 1.5 µg of peptides were analyzed by liquid chromatography-mass spectrometry using an Orbitrap Exploris 480 (Thermo Fisher Scientific), in DIA mode (44). Two blanks were run between each sample. Mass spectrometry data were processed using Spectronaut version 17 using the stringent settings described in reference 45, including mouse and *C. albicans* FASTA files: *Mus musculus* SwissProt canonical with isoforms (February 2022) database and the *C. albicans* TrEMBL (May 2023) database. Protein copy number was determined using Perseus software based on normalization using the histone ruler method (46, 47). Log2 fold change between the wild-type and knockout samples was calculated, and significance was determined by two-tailed *t*-tests on log-transformed data. Missing values were left blank. Enrichment analysis was carried out using ShinyGo V.0.8 (https://bioinformatics.sdstate.edu/go/) (48). Heat maps were generated using Morpheus (https://software.broadinstitute.org/morpheus). A list of genes that showed a threefold induction in mRNA following type 1 interferon treatment was generated based on the data reported in reference 26. A heatmap was generated using Morpheus (broadinstitute.org).

### Seahorse metabolic assay

Macrophages (6 × 10^5^) were seeded in a Seahorse Extracellular Flux 24-well (XF24) culture plate with or without heat-killed *C. albicans* at an MOI of 2 and incubated overnight. The probe plates were pretreated, and the calibration solution was prepared following the manufacturer’s protocol and placed in a CO_2_-free incubator overnight. Cells were washed three times with Seahorse XF Base Medium (Agilent) containing 5 mM glucose and 10 mM HEPES, with pH adjusted to 7.4. Cells were then incubated in this media at 37°C without CO2 and analysis performed using the Seahorse XF Cell Mito Stress Test Kit (Agilent, 103015-100) using the Extracellular Flux Analyzer (Agilent Technologies, Santa Clara, CA) as per the manufacturer’s protocol. The assay involved five measurement cycles of basal OCR and ECAR, followed by sequential injections of 1 µM oligomycin to determine the ATP-linked respiration (oligomycin sensitivity) and proton leak (oligomycin resistance); 1 µM carbonyl cyanide 4-trifluoromethoxy phenylhydrazone to determine the maximal respiration; and then a mix of 1 µM rotenone and 2 µM antimycin A to measure the non-mitochondrial respiration. After each of these injections, four measurement cycles of OCR and ECAR were taken. Real-time metabolic changes in cells were calculated using the Seahorse MITO Stress Test Report Generator on the Seahorse Wave software (Agilent), generating an output for basal and maximal respiration, ATP production, and spare respiratory capacity determined from the OCR readings of the XF24 plate reader. Basal glycolysis and glycolytic capacity were also determined by taking the values of the glycolysis before and after the injection of oligomycin, respectively. All data were normalized to the total microgram of protein per well, determined by a protein assay kit (Bio-Rad).

### Macrophage viability assay

A LIVE/DEAD Cell Imaging Kit (488/570) (Thermo Fisher, R37601) was used to determine the viability of cells following the incubation with *C. albicans* as per the manufacturer’s protocol. Live Green and Dead Red solutions were mixed, and 100 µL was added to each well of macrophages (1 × 10^5^ cells per well) tested and incubated at room temperature for 15 min. Cells were subsequently visualized under a fluorescence microscope at both 488 and 570 nm to capture images of stained live and dead cells, respectively. In selected experiments, including live cell imaging, 0.6 mM DRAQ7 (Abcam) instead of LIVE/DEAD imaging solutions was added for tracking macrophage membrane permeabilization and assessed using fluorescence.

### Data analysis

Statistical analysis was performed in RStudio v.1.1.453 and GraphPad Prism v.5.04, and graphs were created in GraphPad Prism v.5.04. Comparisons between groups were performed using Student’s *t*-tests or, if not normally distributed, by Mann-Whitney U-test. Multiple groups were compared by one-way analysis of variance (ANOVA), or two-way ANOVA was used for comparison among multiple groups with two-factor design, considering the interaction between two factors. Survival analysis was performed using the log-rank test. Data were log-transformed or non-parametric equivalents (Kruskal-Wallis) were used where appropriate. Pearson correlation and linear regression were used in correlation assessment. *P*-values <0.05 were considered statistically significant. For most figures, mean values ± SEM are shown.

## Data Availability

The mass spectrometry proteomics data have been deposited to the ProteomeXchange Consortium via the PRIDE (49) partner repository with the data set identifier PXD057300.

## SUPPLEMENTAL MATERIAL

Supplemental material mbio.01516-25-s0001.docx Table S1 caption, Table S2, and Figures S1 to S8.

Table S1 mbio.01516-25-s0002.xlsx Proteomic analysis of samples from macrophage preparations from wild-type and PTP1B knockout mice.

ASM does not own the copyrights to Supplemental Material that may be linked to, or accessed through, an article. The authors have granted ASM a non-exclusive, world-wide license to publish the Supplemental Material files. Please contact the corresponding author directly for reuse.
