# Supplementary material for: PTP1B deficiency in myeloid cells increases susceptibility to Candida albicans systemic infection by modulating antifungal immunity
Source: mBio. 2025 Aug 29;16(10):e01516-25. doi: 10.1128/mbio.01516-25 (PMC12505889; doi:10.1128/mbio.01516-25)
Supplement: Supplemental material — Table S1 caption, Table S2, and Figures S1 to S8. [file mbio.01516-25-s0001.docx]

Manuscript ID mBio01516-25 **PTP1B deficiency in myeloid cells increases susceptibility to *Candida albicans* systemic infection by modulating antifungal immunity, Allen et al.**

**Supplemental Information Tables and Figures**

**Supplemental Tables**

**Table S1**

See excel file showing proteomic analysis of samples from independent macrophage preparations from 4 mice per genotype (wild type and PTP1B knock out).

| Gene Name | Log_2_FC(PTP1B^-/-^/WT) | -Log_10_(p-value) |
| --- | --- | --- |
| Zbp1 | 1.06236 | 3.547747 |
| Ifit2 | 0.919536 | 3.53354 |
| Oasl1 | 1.264697 | 3.512153 |
| Stat1 | 0.589183 | 3.468441 |
| Fcgr1 | 0.419729 | 3.34476 |
| Pyhin1 | 0.908886 | 3.339133 |
| Pnp | 0.301476 | 3.282141 |
| Oas3 | 0.541834 | 3.24372 |
| Ifi35 | 0.426677 | 3.183803 |
| Sp100 | 0.578233 | 3.169821 |
| Ifit1 | 0.946653 | 3.160254 |
| Ifi44 | 0.748214 | 3.078574 |
| Irgm2 | 0.737468 | 3.041656 |
| Gbp6 | 0.907692 | 2.925418 |
| Irgm1 | 0.355402 | 2.897664 |
| Cmpk2 | 0.785576 | 2.897591 |
| Rnf213 | 0.420551 | 2.798001 |
| Stat2 | 0.487107 | 2.745131 |
| Phf11 | 0.908931 | 2.523086 |
| Samd9l | 0.317682 | 2.452284 |
| Parp14 | 0.292381 | 2.421087 |
| Ifit3 | 1.033777 | 2.407576 |
| Ube2l6 | 0.421807 | 2.309729 |
| Dtx3l | 0.459267 | 2.24965 |
| Naa25 | 0.150605 | 2.242488 |
| Ifih1 | 0.471423 | 2.225932 |
| Isg20 | 1.022583 | 2.179234 |
| Oas1a | 0.328066 | 2.137402 |
| Nampt | 0.136376 | 2.056793 |
| Trim30a | 0.369131 | 2.049918 |
| Eif2ak2 | 0.389876 | 2.000254 |
| Oas2 | 0.619481 | 1.947567 |
| Parp9 | 0.347592 | 1.9175 |
| Nmi | 0.457099 | 1.895635 |
| Dhx58 | 0.55326 | 1.864092 |
| Slfn1 | 1.987158 | 1.797265 |
| Xaf1 | 0.486667 | 1.748713 |
| Ctla2b | 0.442021 | 1.69395 |
| Tor3a | 0.221736 | 1.673839 |
| Usp18 | 0.503113 | 1.67185 |
| Trafd1 | 0.250658 | 1.623818 |
| Rasgef1b | -0.71336 | 1.611123 |
| Trim21 | 0.198698 | 1.576618 |
| Herc6 | 0.443321 | 1.522366 |
| Slfn5 | 0.250436 | 1.38706 |
| Rtp4 | 0.668016 | 1.307377 |
| Oasl2 | 0.407311 | 1.235199 |
| Iigp1 | 2.198881 | 1.210918 |
| Irf7 | -1.41014 | 0.951707 |
| Znfx1 | 0.094941 | 0.922555 |
| Dck | 0.426735 | 0.895605 |
| Gbp5 | 0.382379 | 0.786857 |
| Cxcl10 | 0.308695 | 0.783797 |
| Nfxl1 | 0.215051 | 0.558736 |
| Ifi204 | 0.166566 | 0.55449 |
| Csf1 | 0.351334 | 0.48939 |
| Slfn9 | 0.105237 | 0.396212 |
| Clec2d | 0.079078 | 0.353907 |
| Ifi203 | 0.284917 | 0.353377 |
| Mitd1 | -0.16121 | 0.301439 |
| Cd274 | -0.29135 | 0.240662 |
| Ddx60 | -4.86313 | 0.211966 |
| Rsad2 | 0.086431 | 0.186914 |
| Daxx | 0.070565 | 0.179718 |
| Gch1 | 0.029647 | 0.086593 |
| Dcp2 | 0.21198 | 0.063309 |
| Parp12 | -0.01422 | 0.058103 |

**Table S2 Type 1 interferon genes changed in PTP1B^-/-^ and wild type macrophages after *C. albicans* infection.** Log2 fold change of differentially regulated proteins in PTP1B ^-/-^ versus wild type macrophages following 8 hours *C. albicans* infection and -log10 p values of the change of each.

**Supplemental Figures (S1-S8)**
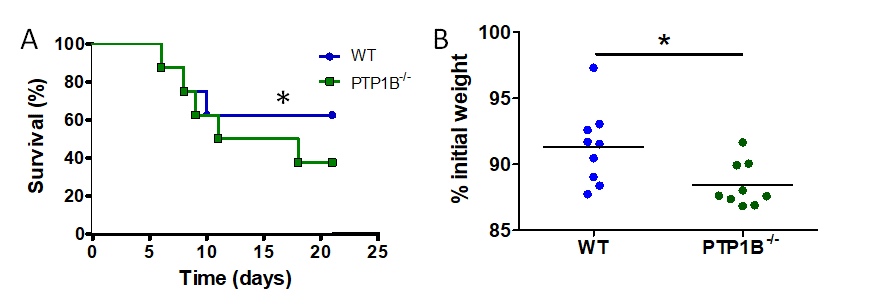


**Fig S1. PTP1B regulates antifungal immunity in vivo. (A)** Survival curve of WT and LysM PTP1B^-/-^ mice after *C. albicans* challenge. WT and LysM PTP1B^-/-^ mice (8 mice/group) were infected intravenously with 1x10^5^ CFU of *C. albicans* and percentage survival was calculated over the time period indicated. * p<0.05, Mantel-Cox log rank test. (B) WT and LysM PTP1B^-/-^ mice were infected with 2.5x10^5^ CFU of *C. albicans* and sacrificed 24h post infection (WT (n=9) and LysM PTP1B^-/-^ (n=9) Body weights were recorded for each mouse following infection and expressed as percentage of initial total body weight prior to infection. * P<0.05, by student *t* test.

**Fig. S2. Cytokine levels correlate with fungal burdens in kidney.** Correlations between cytokine levels and fungal burdens (CFU/g) in kidney of WT (blue) and LysM PTP1B-/- (green) mice at 24h post systemic infection with *Candida albicans*. Pearson correlation and linear regression were used for statistical analysis

**
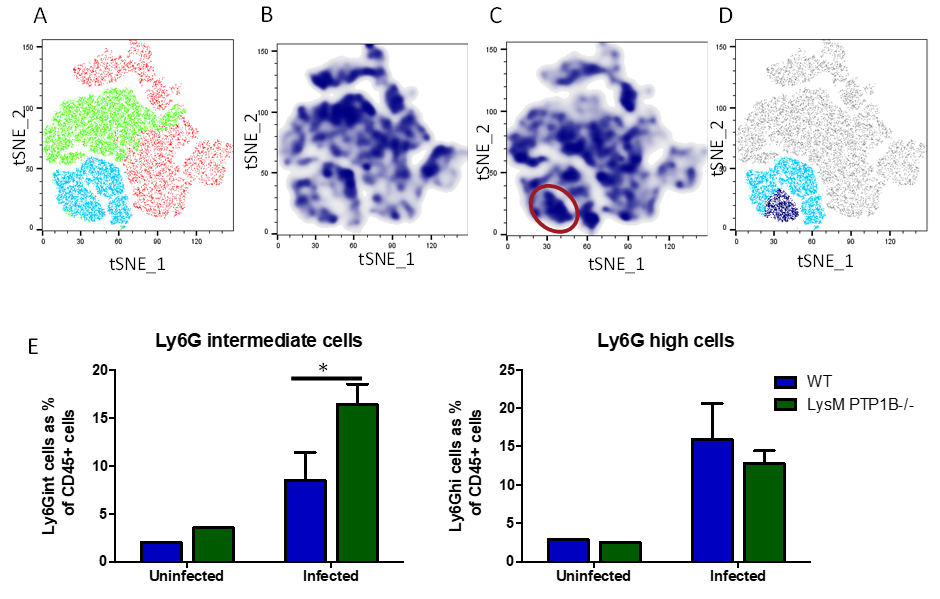
**

**Fig S3. Neutrophils isolated from WT and LysM PTP1B knock out mice kidneys show differences in phenotype. (**A) tSNE diagram for single cell samples of flow cytometry detected kidney cells representing different cell clusters with green representing CD45 positive Ly6C high monocytes, blue representing CD45 positive Ly6G high neutrophils and red indicating ungated cells. (B) tSNE diagram of all cells detected from WT mice and (C) LysM PTP1B knock out mice. (D) tSNE diagram of CD45 positive Ly6G positive cells identified by flow cytometry. Blue represents CD45 positive, Ly6G positive cells similar in WT and LysM PTP1B^-/-^ cells and purple represents the greater abundance of CD45 positive Ly6G intermediate cells in LysM PTP1B^-/-^ mouse kidney. E The percentage of CD45 positive Ly6G intermediate and Ly6G high cells isolated from kidneys of WT and LysM PTP1B^-/-^ mice; uninfected n=1; infected n=3; * p<0.05. Two-way ANOVA with Tukey post hoc correction.


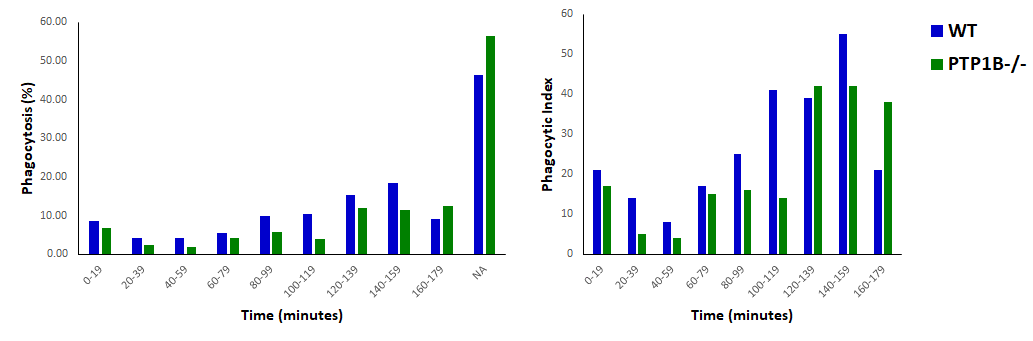
**Fig S4. LysM PTP1B macrophages show dampened uptake of *C. albicans* over time**. Real-time kinetics of *C. albicans* uptake by BMDM. WT or LysM PTP1B^-/-^ BMDM cells were seeded in an 8-chamber μ-slide at a density of 5 x 10^4^ cells per chamber. Cells were co-incubated for 3 hours with *C. albicans* (MOI=1). Phagocytosis kinetics of all macrophages were monitored in designated chamber-points for full engulfment of yeast cells through live-cell spinning disk microscopy (8 points per cell-type). Full engulfment was defined as the time point at which a single *C. albicans* cell is fully enclosed within the macrophage after initial cell wall to cell membrane contact. Representative example of average percentage of phagocytosing cells (A) and phagocytic index (B) for each 20-minute time interval. NA= number of macrophages that did not undergo phagocytosis over the time period of the experiment.


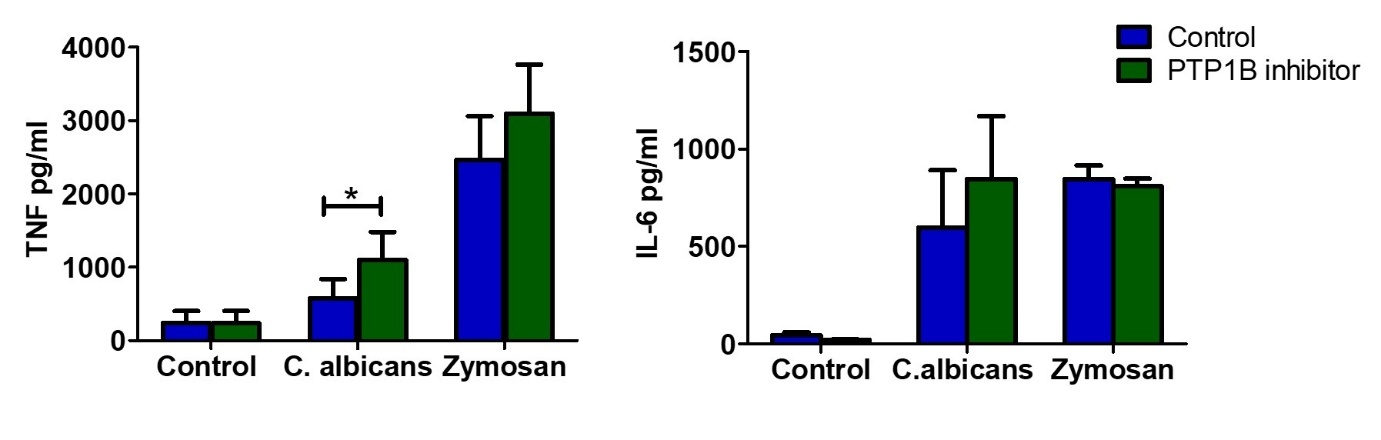


**Fig S5. *C. albicans* stimulated human monocyte derived macrophages show increased TNF-alpha but not IL-6 production following PTP1B inhibition.** Human monocyte derived macrophages with and without pre-treatment with pharmacological PTP1B inhibitor MSI 1436 (0.7 µM) were left unstimulated (control) or stimulated with heat killed *C.albicans*, MOI=5 or zymosan 100 µg/ml for 4 hours and TNF-alpha and IL-6 in supernatants determined by ELISA n=4-8 individual preparations per group. * p<0.05, Two way ANOVA with Tukey post hoc correction.


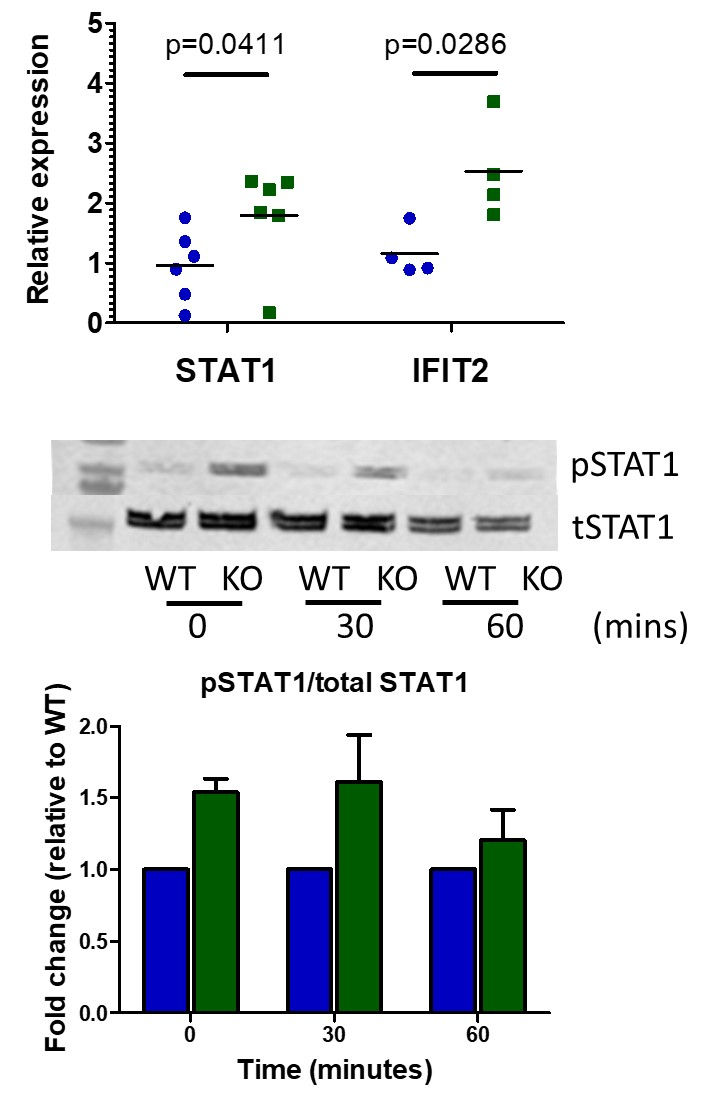
 A

B

C

**Fig S6. Type I interferon signature genes and phosphorylation of STAT1 are enhanced in *Candida albicans* infected mouse bone marrow derived macrophages (BMDM) or kidney from LysM PTP1B^-/-^ mice, respectively.** Expression levels of chemokines *stat1* and *ifit2*, as determined by real-time RT-qPCR, in wild type (WT) and LysM PTP1B^-/-^ mice kidneys isolated 24 hours post infection with *C. albicans* (A). Results were normalized to the expression of the housekeeping genes and are presented as fold change relative to median expression in WT. * p<0.05, Students t-test. Cell lysates from BMDM from wild type and LysM PTP1B^-/-^ mice were left unstimulated or activated by *C. albicans* for 30 or 60 minutes and subject to immunoblotting with phospho STAT1 antibodies then membranes stripped and re-probed with monoclonal antibodies against total STAT1 protein (B). Band intensity was determined and ratios of phospho to total STAT1 calculated. pSTAT1/total STAT1 in LysM PTP1B^-/-^ cells were normalised to that of wild type cells which had a relative value as 1 (C). Shown as mean ± SEM of 4 independent BMDM preparations.

**
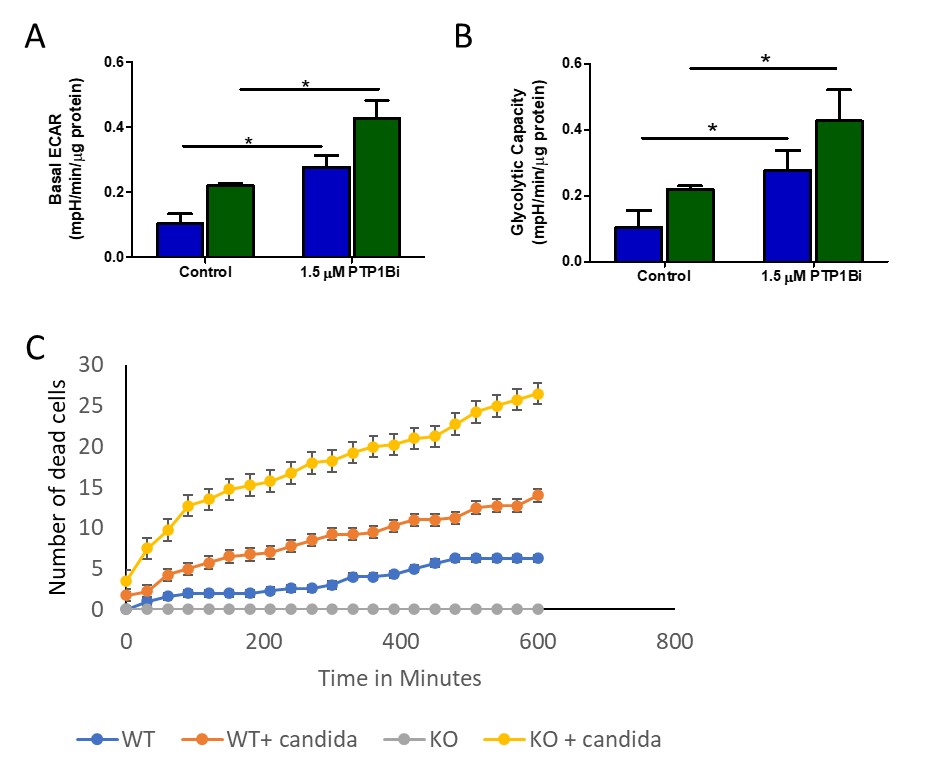
**

**Fig. S7. Inhibition of macrophage PTP1B alters metabolic activity and cell viability.** Metabolic characteristics of human macrophages treated with PTP1B inhibitor in the presence and absence of *C. albicans*. Macrophages were treated without or with PTP1B inhibitor, MSI 1436 (1.5 µM) for 24 hours, then left uninfected or subjected to heat killed *C. albicans* (MOI=1). Analysis with a Seahorse XF24 Analyzer generated outputs for (A) extracellular acidification rate (ECAR) and (B) Glycolytic Capacity. Data represents the mean ± SEM; n=3. * = p<0.05, determined using a one-way ANOVA with Tukey’s multiple comparisons post hoc test. (E) Real-time kinetics of cell death in WT and PTP1B^-/-^ BMDM infected with *C. albicans*. BMDM cells were seeded in an 8-chamber μ-slide at a density of 5 x 10^4^ cells per chamber and co-incubated with or without *C. albicans* (MOI=1) along with 0.6 mm DRAQ7 which fluoresces red upon cell death. The death kinetics of all macrophages were monitored at designated chamber-points using live-cell spinning disk microscopy (4 points per cell-type) with images captured every 2 minutes over the time course of the experiment. Shown are mean values and standard deviations (SD) from a representative experiment.


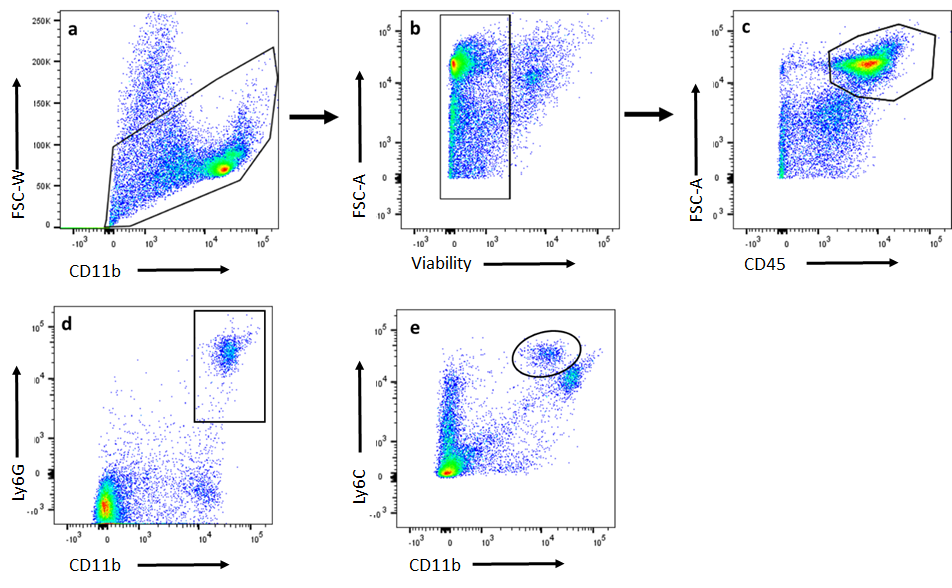


**Fig. S8. Gating strategy for flow cytometry of kidney and spleen cells.** Kidney and spleen cells were initially gated on singlets (a), then live cells, which were defined as cells negative for eFluor780 (APC-Cy7) fluorescence (b). Leucocytes were gated via FSC-A and CD45 (Qdot 655) expression (c). Of the CD45+ leucocytes, neutrophils were defined as CD45+ CD11b+ Ly6G+ (d), and inflammatory monocytes were defined as CD45+ CD11b+ Ly6C^hi^ (e).
